# The Tumor Immune Microenvironment in Primary CNS Neoplasms: A Review of Current Knowledge and Therapeutic Approaches

**DOI:** 10.3390/ijms24032020

**Published:** 2023-01-19

**Authors:** Anita L. Kalluri, Pavan P. Shah, Michael Lim

**Affiliations:** 1 Department of Neurosurgery, The Johns Hopkins University School of Medicine, Baltimore, MD 21205, USA; 2 Department of Neurosurgery, Stanford University School of Medicine, Stanford, CA 94305, USA

**Keywords:** brain cancer, tumor microenvironment, immunotherapy

## Abstract

Primary CNS neoplasms are responsible for considerable mortality and morbidity, and many therapies directed at primary brain tumors have proven unsuccessful despite their success in preclinical studies. Recently, the tumor immune microenvironment has emerged as a critical aspect of primary CNS neoplasms that may affect their malignancy, prognosis, and response to therapy across patients and tumor grades. This review covers the tumor microenvironment of various primary CNS neoplasms, with a focus on glioblastoma and meningioma. Additionally, current therapeutic strategies based on elements of the tumor microenvironment, including checkpoint inhibitor therapy and immunotherapeutic vaccines, are discussed.

## 1. Introduction

Primary central nervous system (CNS) neoplasms represent the 13th most common type of cancer worldwide and are responsible for over 250,000 deaths annually [1]. CNS neoplasms arise from a number of progenitor cell types, with the most common malignancies being meningiomas and gliomas. While CNS malignancies are responsible for considerable morbidity and mortality, the development of novel therapies for these tumors has proven to be difficult due to challenges such as the blood–brain barrier, and promising pre-clinical treatments have often failed to produce meaningful differences in clinical trials.

In recent years, as our understanding of the diversity and complexity of tumors has evolved, there has been an increasing focus on the sub-characterization of tumors to optimize therapy selection, as well as the identification of novel therapeutic targets to improve outcomes for otherwise treatment-resistant cancers. One particular tumor feature that has emerged at the forefront of these efforts is the immune microenvironment, defined by the specific mix of immune cells associated with a tumor and the extensive crosstalk that occurs between tumor cells and these immune cells.

While the tumor microenvironment (TME) is known to be an important factor in the pathogenesis of systemic malignancies, the CNS was historically considered to have a paucity of immune activity [2]. However, recent developments have shown that the CNS contains a distinctive immune environment that exerts both immunosuppressive and anti-tumor effects on CNS neoplasms throughout the course of their pathogenesis. This review summarizes our current understanding of the TMEs of various primary brain tumors, focusing primarily on high and low-grade gliomas and meningiomas. Additionally, we discuss various therapeutic strategies that harness or target the unique TME elements present in CNS malignancies.

## 2. Tumor Microenvironment of Various Intracranial Primary Brain Tumors

### 2.1. Glioblastoma

**Figure 1:**
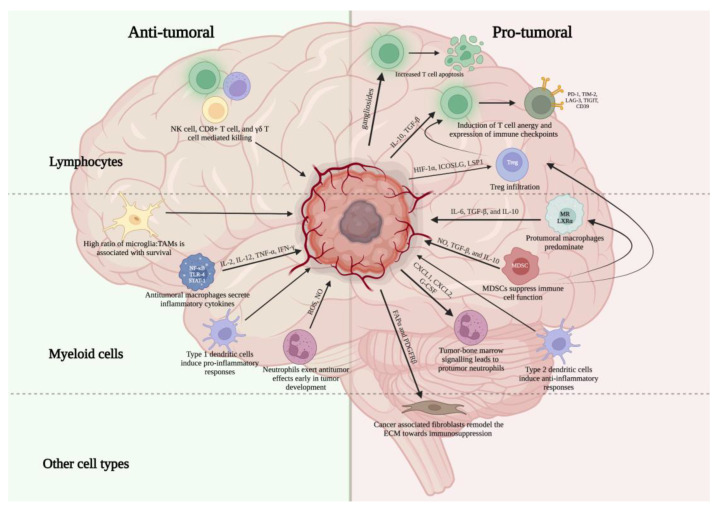
Overview of key pro- and anti-tumor immune cell populations within the glioblastoma microenvironment. Created with Biorender.com.

Glioblastoma (GBM) is the most malignant form of glioma and carries a very poor prognosis, with a median survival of 15 months [3]. GBM is notable for its particularly immunosuppressive microenvironment, leading to tumor evasion of immune responses and the failure of a number of attempted therapies. This immunosuppressive environment is complex and multifactorial, resulting from the recruitment of immunosuppressive cell populations, high levels of lymphocyte exhaustion, immunometabolism alterations in the local environment, and structural changes in the local tumor environment. Key cell types within the immune microenvironment of GBM include tumor infiltrating lymphocytes (TILs), natural killer (NK) cells, tumor associated macrophages (TAMs), microglia, myeloid-derived suppressor cells (MDSCs), neutrophils, dendritic cells, and fibroblasts [4] (Table 1, Figure 1).

#### 2.1.1. Key Immune Cell Populations

##### Lymphoid Cells

Cytotoxic T cell dysfunction contributes to high-grade glioma pathogenesis. Compared to healthy controls, a higher proportion of CD4+ and CD8+ T cells infiltrating GBM tumors are apoptotic, particularly activated T cells expressing the Fas ligand [5]. Numerous mechanisms, including the induction of T cell apoptosis via the secretion of gangliosides by GBM tumor cells and the activation of TAMs that disrupt T cell function, may underlie GBM-driven T cell dysfunction and apoptosis [70,71]. Additionally, patients with GBM have been shown to have limited T-cell clone diversity compared to healthy patients [6]. The TME contains a number of immunosuppressive cytokines that may be responsible for T cell dysfunction. For example, glioma cells secrete or induce secretion of the immunosuppressive cytokines IL-10 and TGF-β, which can increase the threshold for T cell activation, and directly suppress their anti-tumor activity, allowing for tumor proliferation [7]. In addition to tumor cells, Ravi et al. demonstrated that *HMOX1+* myeloid cells can also secrete IL-10, causing T cell dysfunction and immune evasion [48]. Paradoxically, IL-10 has also been demonstrated to have anti-glioma effects in a mouse model when expressed in conjunction with other cytokines, such as IL-2 [7,72]. The contradictory roles of IL-10 are likely contingent upon environmental cues and individual tumor characteristics, further complicating potential therapeutic interventions aimed at altering IL-10 pathways. Notably, the cytokine TGF-β also has a significant immunosuppressive role and has been shown to inhibit the cytotoxic abilities of various immune cell types, including T cells [7,73]. In preclinical models of glioma, siRNA silencing of TGF-β led to increased immune cell lysis of glioma. Furthermore, the inhibition of the TGF-β receptor in a murine model of glioma led to higher levels of CD8+ T cells and increased survival [74,75].

In addition to an increased barrier for initial activation, T cell overstimulation and exhaustion is also a defining feature of the GBM immune microenvironment. Tumor infiltrating lymphocytes (TILs) in GBM have been shown to express PD-1, a marker of exhaustion, at higher rates than peripheral T cells in the bloodstream [8]. Glioma-associated IL-10 upregulates the expression of PD-L1 on TAMs and peripheral monocytes, which can then bind to and stimulate PD-1 on TILs, leading to T cell anergy [9,10]. Notably, Davidson et al. found that PD-1+ T cells in patients with malignant glioma have decreased diversity compared to PD-1- T cells [8]. Woroniecka et al. further characterized T cell exhaustion in glioblastoma, reporting that multiple immune checkpoints, in addition to PD-1, such as TIM-2, LAG-3, TIGIT, and CD39, were expressed by TILs [76]. These TILs secreted lower levels of immune-stimulating factors, including IFN-γ, IL-2, and TNF-α, than TILs from control blood or patient blood [76]. Notably, TILs that were positive for PD-1, TIM-3, and LAG-3 were less functional than PD-1 single positive cells, demonstrating a joint role for these immune checkpoint molecules in inducing T cell exhaustion in GBM [76]. Overall, cytotoxic T cell dysfunction in the GBM TME contributes significantly to tumor immune escape and results from increased barriers to activation, decreased anti-tumor function, and high levels of exhaustion.

FOXP3+ T regulatory cell (Treg) enrichment has also been observed in glioblastoma and other high-grade tumor types [11,12]. Treg infiltration of the GBM TME may be mediated by numerous pathways, including hypoxia inducible factor 1α (HIF-1α), inducible T cell co-stimulator ligand (ICOSLG), lymphocyte-specific protein 1 (LSP1), and tolerogenic IDO-1+ dendritic cells [77,78,79,80]. While these cells likely contribute to immunosuppressive tumor environments, studies have failed to show a significant correlation between Treg presence and GBM patient survival [13,14,15]. Antunes et al. suggest that this may be due to the presence of distinct Treg subtypes, such as tumor-specific subtypes that are not present in the periphery, which differentially contribute to the immunosuppressive environment in GBM [4,81]. For example, CCR8 is a marker of multiclonal Tregs in tumors, which may play an immunosuppressive role, and the targeted depletion of CCR8-expressing cells may have potent anti-tumor effects [82]. Furthermore, Treg accumulation in GBM may be due to the interaction of CCR4, which is highly expressed on Tregs, and CCL22, which is a CCR4 ligand secreted by GBM tumor cells [13]. Further studies are needed to characterize the immunosuppressive contributions of different Treg subsets to the TME in GBM and identify potential therapeutic targets.

Recent studies have also examined the role of γδ T cells in GBM pathogenesis and prognosis [77]. γδ T cells are unique in that they can recognize and bind to antigens in an MHC-independent fashion. Lee et al. identified γδ T cells located near TAMs in GBM tumor tissue from four patients and reported that γδ T cell immune-related gene expression was similar to that of cytotoxic T cells and anti-tumor macrophages, suggesting a potential anti-tumor role [16]. Additionally, γδ T cells can eliminate GBM cells in a murine model via a granzyme-mediated mechanism when sensitized by the cytokine IL-21 [17]. Notably, Chauvin et al. reported that γδ T cells eliminate mesenchymal GBM cells via the NKG2D pathway, and the bisphosphonates zoledronate and minodronate have been shown to exert anti-tumor effects in vitro via the stimulation of γδ T cells [18,19,20]. Given that γδ T cells may be depleted in GBM patients compared to healthy patients, increasing γδ T cell infiltration and activation may be a promising immunotherapeutic strategy [21].

Natural killer (NK) cells have also been characterized as a component of the GBM TME. A number of studies investigating the immune gene expression profile in gliomas have reported that activated NK cells are associated with a better prognosis for glioma patients [22,23,24,25]. Interestingly, however, one study also reported that activated NK cells were predictive of malignant transformation of low-grade glioma [25,83]. Studies in mice support the finding that NK cells have cytotoxic effects against GBM tumor cells, with one study reporting that the inactivation of NK cells promoted lung metastases in a murine GBM model [84,85]. Furthermore, a study investigating the relationship between immune phenotypes and GBM patient prognosis found that activated NK cells were associated with improved patient survival [86]. However, the anti-tumor effect of NK cells may differ according to the level of tumor differentiation. Kozlawska et al. demonstrated that while activated NK cells have a demonstrated cytotoxic effect against GBM stem cells, NK cells may also contribute to GBM differentiation via interferon gamma (IFN-γ) signaling [26]. While differentiated GBM tumor cells are less susceptible to NK-cell mediated killing, they also have limited growth potential and may be more susceptible to chemotherapy, suggesting a role for a multimodal therapeutic approach [26,27]. A potential therapeutic role for NK cells is supported by reports that both intracranially and systemically delivered NK cells can home to GBM tumor and exert a cytotoxic effect in a murine model [87].

##### Myeloid Cells

Myeloid cells are the largest immune component of the GBM TME and play a significant role in both the overall immune response and efficacy of therapies for GBM patients [88]. Overall, the myeloid compartment of the TME is viewed as immunosuppressive, and a number of emerging therapeutic pathways are aimed at either reducing this immunosuppression or re-programming myeloid cells to have anti-tumor responses [89]. However, the myeloid compartment of the GBM TME is actually made up of a number of different cell types with both pro- and anti-tumor functions, including macrophages (TAMs), microglia, myeloid-derived suppressor cells (MDSCs), neutrophils, and dendritic cells.

TAMs are a major type of immune cell within the GBM TME and exert both pro-tumor and anti-tumor effects [28]. Historically, TAMs have been categorized as either M1 (pro-inflammatory) or M2 (anti-inflammatory), with the former suppressing tumor progression and the latter contributing to immunosuppressive environments that promote tumor growth. For example, M1 macrophages were thought to release inflammatory cytokines (such as IL-2, IL-12, IFN-γ and TNF-α), express a distinct set of metalloproteinases, and suppress angiogenesis, while M2 macrophages were through to release immunosuppressive cytokines (such as IL-6, IL-10 and TGF-β) and promote angiogenesis [7,90,91,92,93,94].

However, macrophage polarization in CNS tumors is likely more complex than previously thought, with recent studies suggesting that the M1/M2 stratification may be oversimplified [28,29,30,31]. In fact, recent single-cell profiling studies in gliomas have uncovered a diversity of myeloid cell populations, none of which approximate the classic M1 or M2 phenotypes [32,33,34]. Instead, TAMs develop disease-specific alterations and are capable of adapting to various immunogenic cues in the local microenvironment (such as chemokines, cytokines, metabolic changes, microRNA, oxygen availability, and pH) [35,36,37]. For example, Friebel et al. performed single-cell mapping of immune cells in gliomas and reported that the signature trajectory of TAMs was driven by the pathogenic insult, i.e., tumor type, rather than by CNS tissue [32]. Lin et al. also reported the presence of monocytes with stem cell-like properties in tumor tissues that may exist and proliferate in a self-renewing state without differentiating, and numerous studies have identified heterogeneous populations of macrophages expressing markers that were classically associated with both the M1 and M2 phenotype [38,39,40,41,42]. Furthermore, Zilionis et al. utilized single cell transcriptomics to analyze tumor-invading myeloid cells and defined 14 different monocyte/macrophage transcriptional states [95]. Thus, while past studies have utilized the M1/M2 distinction, future studies should focus on analyzing macrophages by genotype, the produced cytokine, or functionality.

Given the evolving views regarding the validity of M1 vs. M2 polarization, researchers have also begun exploring other ways to define sub-populations of TAMs within the GBM TME. For example, a number of studies have characterized the developmental origins of TAMs in GBM. Chen et al. reported that 85% of TAMs were monocyte-derived cells from the bone marrow, whereas 15% were resident microglia [42]. Furthermore, they found that monocyte-derived macrophages and resident microglia had differing localization and gene-expression patterns, suggesting different contributory roles of these TAM subtypes to GBM pathogenesis and progression [42,96]. Woolf et al. investigated the role of microglia in GBM via single-cell image analysis of resected tissues and found that a greater ratio of microglia to TAMs was correlated with an increase in patient survival [43]. Overall, TAMs derived from bone marrow seem to express immunosuppressive proteins at higher levels than microglia when stimulated in vitro, but in vivo studies remain inconclusive regarding differences in function due to the difficulty in reliably characterizing and observing these cell populations [97]. Thus, modulating the ability of monocyte-derived macrophages to migrate to the CNS may present a potential therapeutic avenue. For example, monocyte migration is driven by the monocyte chemoattractant protein CCL2 via the CCR2 receptor, and CCL2 downregulation resulted in reduced monocyte migration and prolonged survival in a murine model of glioblastoma [98].

Studies specifically investigating resident microglia in glioblastoma suggest that these “professional phagocytes” of the central nervous system do not successfully phagocytose invading glioma cells. This may be due to increased expression of CD47, an anti-phagocytotic surface protein, on tumor cells [44]. A high level of leucine rich repeats and immunoglobulin like domains 2 (LRIG2) expression, which is linked with GBM progression and poor prognosis, has been shown to increase CD47 expression, and CD47 expression has been shown to be associated with a higher tumor grade and worse clinical outcomes [99,100]. Furthermore, Hutter et al. and Gholamin et al. reported that anti-CD47 reduced tumor growth in orthotopic xenograft models of glioblastoma and various pediatric brain tumor types, respectively [45,46].

Researchers have also begun investigating the signaling pathways altered in pro-tumor microglia. For example, a tumorigenic microglial phenotype may also be induced by the upregulation of the mammalian target of rapamycin (mTOR) pathway in GBM and by the downregulation of microglial sensome genes, such as sialic acid-binding immunoglobulin-like lectin-H (Siglech); these are critical for sensing tumor cells and their byproducts [101,102]. Along with phagocytic dysfunction, tumor-induced microglial changes may also inhibit the recruitment of effector immune cells, thus promoting immune evasion [101].

Myeloid-derived suppressor cells (MDSCs) also contribute significantly to the overall immunosuppressive environment of glioblastoma [47,103]. MDSCs mediate immunosuppression by suppressing the function of cytotoxic T cells, NK cells, anti-inflammatory macrophages, and dendritic cells through the production of nitric oxide and various immunosuppressive cytokines, such as IL-10 and TGF-β [48,49,50] Further, they promote the recruitment of Tregs, B cells and M2 macrophages, which contribute to high-grade glioma progression [49,51]. Lee-Chang et al. also investigated the role of MDSCs in promoting B-cell mediated immunosuppression in GBM and reported that MDSCs promote B cell regulatory function via the transfer of membrane-bound PD-L1, which gives regulatory B cells the ability to mediate immunosuppression via the inhibition of CD8+ T cell activation [51]. Studies have also reported both increased numbers of monocytic-MDSCs in the peripheral blood of patients with high-grade gliomas and higher levels of activation in these MDSCs compared to healthy controls [104,105]. Furthermore, tumor infiltration and immunosuppression by MDSCs, which are dependent on tumor cell expression of macrophage migration inhibitory factor (MIF), have been directly associated with poor prognosis [104]. Given these findings, MDSCs may have a potential future role as biomarkers for GBM detection and prognosis [104,106].

Neutrophils exhibit both anti-tumor and pro-tumor phenotypes within the TME [52,53]. Cues secreted by the TME may influence neutrophil polarization; for example, IFN-β induces anti-tumor activity, whereas TGF-β induces pro-tumor activity [52,53,55]. Notably, like TAMS, neutrophils were previously thought to have a binary anti-tumor (N1) or pro-tumor (N2) phenotype, but recent work has demonstrated that they actually have a wider spectrum of activation states [53]. Specifically, at least three subsets of neutrophils have been identified: mature high-density neutrophils (HDNs), immature low-density neutrophils (LDNs), and mature low-density neutrophils [53,107,108,109,110]. HDNs appear to have anti-tumor functions, whereas LDNs may have immunosuppressive functions [53,107,108,109,110]. To better understand the influence of neutrophils on GBM progression, Magod et al. aimed to characterize neutrophil infiltration into the glioma microenvironment temporally and reported that the ability of neutrophils to inhibit tumor growth was lost during GBM tumor progression in a murine model [54]. Furthermore, the authors described a tumor–bone-marrow signaling pathway that resulted in the development of neutrophils with pro-tumor activity from the bone marrow as tumors progressed [54]. Specifically, pro-tumor neutrophils in this study were shown to suppress cytotoxic T cell function and support tumor angiogenesis [54]. By contrast, proinflammatory neutrophils can attract CD8+ T cells via the production of proinflammatory cytokines and chemokines (IL-12, VEGF, TNF-α, GM-CSF, CCL3, CXCL9, and CXCL10) [55,56,57]. Various neutrophil-mediated tumor cell-killing mechanisms have been described, including direct contact and the generation of ROS, NO production, and the induction of calcium influx into tumor cells via the TRPM2 channel [53,111,112,113]. However, some neutrophil-mediated tumor deaths may actually be detrimental to overall patient outcomes. For example, tumor cell ferroptosis, induced by neutrophils via the transfer of granules containing myeloperoxidase, is associated with greater tumor necrosis and poor patient outcomes [58]. Additionally, neutrophils have been implicated in the mechanism of biopsy-associated GBM tumor spreading indirectly via macrophage recruitment and directly via the secretion of soluble factors to induce tumor cell migration [114]. Notably, neutrophil to lymphocyte (NLR) in the periphery may also a prognostic marker for patient outcome. One study demonstrated that a high NLR is associated with tumor progression, decreased survival, and poor prognosis in patients with GBM [115].

Dendritic cells represent another important component of the immune microenvironment in GBM. While not present in normal brain parenchyma, dendritic cells may migrate via afferent lymphatics to the brain in pathological states and present tumor antigens in order to coordinate anti-tumor T cell responses [59,116]. Uncommitted dendritic cells may mature into type-1 or type-2 polarized effector cells, the former of which induce proinflammatory TH1 responses and play a role in anti-tumor immunity [59]. For example, these dendritic cells can present tumor antigens to naïve CD8+ T lymphocytes at tumor-draining lymph nodes and produce cytokines, such as CCL9, CCL10, and IL-12, that enhance the anti-tumor activity of other immune cells, including T cells and NK cells [59,60,61,62,63,64]. The anti-tumor properties of dendritic cells have led to studies investigating their therapeutic applications. For example, Flores et al. reported that hematopoietic stem cell-derived dendritic cells, isolated from GBM, induced anti-tumor immunity in mice when delivered via vaccine. In conjunction with other studies, they also found that dendritic cell presence, induction, and/or activation can enhance the response to immune checkpoint blockade therapy in rodent glioblastoma models [117,118,119]. However, the immunosuppressive environment of high-grade gliomas, including vascular endothelial growth factor (VEGF), prostaglandin E2 (PGE2), and IL-10 expression, may induce a regulatory or tolerant dendritic cell phenotype [65]. Studies suggest that these immunosuppressive factors may suppress dendritic cell maturation via over-expression of the transcription factor Nrf, which regulates the cellular response to oxidative stress [59,120]. Indeed, inhibiting the Nrf pathway has been shown to enhance dendritic cell maturation and promote the cytotoxic T cell response in an in vitro glioma cell preparation [59]. Regulatory dendritic cells, in turn, secrete the immunosuppressive cytokines IL-10 and TGF-β, which promote the activation of Tregs and decrease the recruitment of cytotoxic T cells [65,121,122,123,124].

##### Other Key Cell Populations

Cancer-associated fibroblasts (CAFs) have recently been shown to modify both metabolic and immunologic aspects of the TME in GBM. Through remodeling of the extracellular matrix, CAFs may contribute to the development of an immunosuppressive TME. The expression of proteins involved in the activation of fibroblasts, including fibroblast activation protein α (FAPα) and PDGFRβ, has been shown in both murine models and human GBM [66]. Additionally, Chen et al. established an association between the expression of six CAFs-related genes (*ABCC3*, *CTHRC1*, *MSR1*, *PDLIM1*, *TNFRSF12A*, and *CHI3L2*) and higher risk in gliomas by analyzing the cancer genome atlas and the Chinese glioma genome atlas. The high-risk group included higher tumor grade and poor prognosis [68]. Furthermore, Kim et al. consistently identified small quantities (<1%) of fibroblasts in resected human GBM tumor samples. They also demonstrated that the intracranial implantation of NIH3T3 embryonic fibroblasts led to the development of larger GL261 glioma tumors in a murine model [67]. Additionally, Trylcova et al. demonstrated that CAF-conditioned media increased the chemotactic migration of glioma cells in vitro [69]. These findings support a pro-tumor role for CAFs and identify them as a potential target for future therapies.

#### 2.1.2. Metabolic and Structural Changes within the TME

There are also numerous metabolic alterations within the GBM TME that significantly alter local immune cell functions. Hypoxia is a key feature of the GBM TME, caused primarily by increased demand from tumor cells and errant or insufficient neovascularization. Among these changes is the upregulation of hypoxia inducible factor (HIF) expression, which promotes the differentiation of immunosuppressive cell populations, such as Tregs, and the up-regulation of immune checkpoint ligands, such as PD-L1 [125,126,127]. Hypoxic conditions also alter the cytokine milieu within the TME, increasing the secretion of immunosuppressive cytokines, such as IL-6 and IL-8, by GBM cells [128,129].

In addition to low oxygen levels, reduced glucose levels also promote immunosuppression within the GBM TME. Glycolytic pathways are upregulated by a number of cells within the GBM TME, including tumor cells and metabolically active immunosuppressive cell populations [126,130]. This greatly decreases the available glucose for anti-tumor immune cell populations, which may inhibit a number of key immune functions. For example, studies in other tumor types have demonstrated that decreased glucose availability hinders glycolysis-dependent TCR signaling and may promote exhaustion in T cell populations [131,132]. In addition to the lack of available glucose, the byproducts of glycolysis may also directly exert immunosuppressive effects. Specifically, lactate can cause the apoptosis of CD8+ lymphocytes and NK cells, and local acidification has been shown to promote M2 polarization within macrophage populations [133].

The catabolism of amino acids has also emerged as a key metabolic immunomodulatory factor within the GBM TME. Tryptophan catabolism, through IDO1, IDO2, and TDO2, has been well characterized in the context of GBM and seems to contribute to both immunosuppression and tumor growth [126]. Specifically, the downstream metabolites of tryptophan, such as kynurenine, can promote tumor cell survival and suppress the proliferation of both lymphoid and myeloid cell populations [134]. Interestingly, IDO inhibitor monotherapy did not significantly improve outcomes in pre-clinical studies, but IDO treatment may potentiate radiotherapy [135]. Arginine metabolism is also altered in the context of GBM. GBM cells are dependent on extracellular arginine availability for proliferation, and thus upregulate amino acid transporters and deplete the local environment [126,136]. This arginine depletion directly hinders the function of TILs, which rely on arginine for proliferation and cytotoxic functions [136]. Furthermore, arginine is differentially metabolized by pro-tumor and anti-tumor macrophages. Specifically, M1 polarized TAMs metabolize arginine primarily via nitric oxide synthase and M2 polarized TAMs primarily utilize arginase [137]. Thus, the alteration of arginine availability and metabolism has been proposed as a therapeutic strategy, and arginine deprivation has been noted to potentiate a response to radiotherapy in pre-clinical models [138].

In addition to metabolic alterations, the GBM environment is also characterized by structural changes, such as deficiencies in waste clearance and fluid balance via the glymphatic system. In preclinical studies, impaired lymphatic function has been shown to reduce CSF outflow in glioma-bearing mice [139]. Toh et al. investigated glymphatic function, measured by the index for diffusivity along the perivascular space (ALPS index), in 201 glioma patients and found that IDH-wild type gliomas had a lower ALPS index than IDH-mutant gliomas [140]. They also found that greater volumes of peritumoral edema were associated with a lower ALPS index, suggesting that glymphatic dysfunction may contribute to malignancy [140].

#### 2.1.3. Systemic Immunosuppression

In addition to the profound local immunosuppression within the GBM TME described above, patients with GBM also have significant systemic immunosuppression [141]. Studies have noted that treatment-naïve patients with GBM have decreased systemic lymphocyte levels, seen both as profound CD4+ count depression, as well as atrophic, T cell-depleted lymphoid organs [142,143]. Interestingly, naïve T cells were instead sequestered within the bone marrow, seemingly due to internalization of the key lymphocyte trafficking receptor, S1P1 [143]. Additionally, many widely-used therapies for GBM can worsen this immunosuppression. For example, treatment with dexamethasone further decreases CD4+ counts in GBM patients [144]. Dexamethasone treatment also induces the development of HLA-DR low/- monocytes, which are unable to develop into mature dendritic cells and inhibit T cell proliferation [144]. While the exact mechanisms for the systemic immunosuppression seen in GBM are poorly understood, Ayasoufi et al. demonstrated that serum from mice implanted with GL261 cells hindered T cell proliferation in vitro [142]. Furthermore, they demonstrated that the serum components driving this immunosuppression have a molecular weight of >100 kDa, suggesting that the immunosuppression is not due to circulating steroids, but rather due to larger molecules [142].

### 2.2. Other Gliomas

Key differences have been noted between the TME and immune cell infiltrates of high- and low-grade (IDH-mutant) gliomas, with low-grade gliomas generally having less immune cell infiltration than high-grade gliomas [145] (Table 2). Within low-grade gliomas, the degree of infiltration by specific immune cell types may be of prognostic value. Yang et al. quantitatively characterized the degree of immune cell infiltration (ICI) in low-grade gliomas and found that low ICI scores, associated with higher levels of Tregs, resting NK cells, and M2 macrophages and lower levels of CD8+ T cells, were predictive of poorer survival outcomes [146].

Lymphocyte infiltration has been shown to differ between high- and low-grade gliomas, with studies demonstrating decreased numbers of TILs in low-grade compared to high-grade gliomas [35,145]. Studies investigating the mechanism of lower TIL recruitment have noted that the chemokines CXCL9 and CXCL10, which are implicated in T cell trafficking, are expressed at lower levels in low-grade compared to high-grade gliomas [147,148]. Furthermore, IDH mutations directly increase the production of R-2-hydroxyglutarate (2HG), which may be responsible for the observed decreased CXCL10 levels in low-grade tumors [148].

Immunosuppressive T cells seem to be less prominent in the low-grade glioma TME when compared to high-grade tumors. For example, Garber et al. reported lower levels of PD-1-expressing TILs in low-grade gliomas, suggesting a less inflammatory and subsequently exhausted phenotype in these tumors [149,150,151]. Lower levels of Tregs have also been reported in low-grade gliomas [14,32]. Overall, the differences in T cell characteristics between low- and high-grade gliomas likely contribute significantly to the differential immunosuppression seen between the two tumor types.

IDH mutations may also influence NK cell recruitment in low-grade glioma [152]. Ren et al. reported higher levels of NK cell infiltration in IDH mutant vs. IDH wildtype tumors, which was also associated with improved prognosis in glioma patients [152]. This increase in NK cell infiltration is likely mediated via an IDH-mutation-induced increase in the expression of the chemokine CX3CL1, which is involved in NK cell recruitment [152,153].

While TAMs are the dominant immune cell type in both IDH-mutant and IDH-wild type gliomas, there are important differences in TAM phenotypes among glioma grades. Vidyarthi et al. investigated macrophage polarization in gliomas and reported fewer M2 and more M1 macrophages in low- vs. high-grade gliomas [154]. Furthermore, the dominant developmental pathway for TAMs also differs, with monocyte-derived macrophages dominating over microglia in IDH-wild type gliomas [40,155]. Additionally, tumor progression is associated with a greater macrophage to microglia ratio in the TME [155].

Myeloid cell infiltrate also differs significantly among various low-grade gliomas. For example, astrocytomas are associated with greater numbers of macrophages and microglia than oligodendrogliomas [155]. Differences in immune cell gene expression of different low-grade tumors were noted to be comparable to or even exceed differences between the gene expression programs of oligodendroglioma and astrocytoma tumor cells themselves, suggesting a critical role for the immune microenvironment in determining the unique pathology and prognosis of these tumors despite their shared glial cell origin [155].

### 2.3. Meningioma

Meningiomas are neoplasms that arise from brain or spinal cord meninges [156]. While meningiomas are typically considered benign, these tumors also carry a significant rate of recurrence [157,158]. Unlike tumors arising from brain parenchyma, meningiomas are not protected by the blood–brain barrier or specialized CNS immunoregulatory mechanisms, and are thus more accessible to immune cells in the periphery [159,160]. The immune microenvironment of meningiomas is diverse, featuring lymphocytes (TILs), macrophages (TAMs), microglia, myeloid-derived suppressor cells (MDSCs), dendritic cells, and mast cells [161] (Table 3).

#### 2.3.1. Key Immune Cell Populations

##### Lymphoid Cells

Lymphocytes, predominantly T cells, significantly contribute to the immune cell composition of meningiomas and typically cluster around perivascular spaces [164]. Fang et al. characterized the lymphocytic infiltrate of 28 meningiomas, of which 61% harbored B cells, 28.5% harbored antibody-producing plasma cells, and 100% harbored T cells [164]. High levels of tumor infiltrating CD3^+^CD8^+^FOXP3^−^ lymphocytes were associated with improved progression free survival in high-grade meningiomas [162]. Additionally, Tregs were enriched in high-grade compared to low-grade meningiomas [163], whereas the expression of CXCL16, a T cell and monocyte chemoattractant, was increased in low-grade meningiomas [178]. T cells expressing PD-1, indicative of exhaustion, were shown to be enriched in tumors compared to periphery, suggesting that these cells along with Tregs may contribute to an immunosuppressive microenvironment in meningiomas [164]. PD-L1 and TILs were also specifically noted in neurofibromatosis type 2 meningiomas [179]. Furthermore, Li et al. found that PD-L1-expressing tumor cells were increased in proportion to the WHO tumor grade, suggesting a role for exhaustion in tumor progression [172]. The TME may also influence the likelihood of recurrence, as recurrent meningiomas are characterized by lower numbers of TILs [162].

Notably, meningiomas arising from different locations may exhibit different lymphocytic infiltration profiles. Cavernous sinus meningiomas were found to have decreased overall immune infiltration compared to convexity meningiomas, which may be a result of reduced VEGF signaling in these tumors [180]. Furthermore, Zador et al. reported that skull base meningiomas, which tend to display more benign characteristics, exhibit high levels of γδ T cell infiltration. In contrast, convexity tumors are dominated by mast cells and neutrophils, which may contribute to the more aggressive nature of these tumors [165].

##### Myeloid Cells

TAMs have been reported as the most prevalent immune cell type in the meningioma TME, constituting 18–44% of all cells in meningioma samples [157,166,167,168,169]. A correlation between macrophage infiltration and the expression of the monocyte chemoattractant protein-1 (MCP-1/CCL2) by meningiomas has been demonstrated, suggesting a role for this chemokine in the mechanism of meningioma infiltration by TAMs [181]. Notably, Proctor et al. reported that over 80% of macrophages in meningiomas are of the immunosuppressive M2 phenotype, evidenced by increased levels of CD163 and CD206 expression and low levels of CD80 and CD86 expression [166]. Furthermore, M2 macrophage infiltration has been shown to correlate with tumor size, grade, and recurrence across meningioma subtypes [166,170]. Tumor-associated factors, such as hypoxia, which is characteristic of tumor necrosis, have been noted to promote M2 polarization. In contrast, a higher ratio of M1 to M2 macrophage has been associated with increased progression-free survival [166]. Notably, one study demonstrated that TAMs of meningiomas with a monosomic chromosome 22 deletion had an M1-dominant phenotype [167]. This suggests that the deletion of genes located on this chromosome (such as the *MIF* gene) may have anti-tumoral effects [167]. To better characterize meningioma-associated macrophages and microglia, Grund et al. specifically investigated the infiltration of microglia and macrophages at the border between meningioma tumors and normal brain [171]. Overall, 25% of meningiomas had microglia or macrophagic cells at the tumor–brain border [171]. Furthermore, the presence of these cells was associated with malignant tumor grade and the loss of the pial-glial basement membrane, suggesting that these immune cells may be causally linked to more invasive tumor behavior [171]. Additionally, Woolf et al. investigated myeloid cell populations in resected meningioma tissue and found that microglia were scarce, whereas TAMs were abundant, likely reflecting the non-brain origin of these tumors [43].

MDSCs have also been identified in meningiomas, with increased numbers of MDSCs reported in high-grade, compared to low-grade, meningiomas [172]. Additionally, Pinton et al. observed that while peripheral MDSCs in patients with meningiomas caused minimal immunosuppression, infiltrating MDSCs reduced activated T cell activity [173]. The authors suggest that this may indicate a role for the tumor-induced differentiation of MDSCs into macrophages with immunosuppressive activity [173].

Dendritic cell infiltrate into meningiomas may also offer prognostic insight, though studies are limited. Chen et al. investigated the association between immune cell infiltration profiles and survival in meningioma patients and found that lower levels of dendritic cell infiltration were associated with better survival outcomes [174]. The authors suggest that this survival advantage may be due to a reduction in B-cell receptor signaling in tumors with greater dendritic cell infiltration [174,182].

##### Other Key Immune Cell Populations

Mast cells are also a prominent cell type in the TME of meningiomas. Immunohistochemical staining for tryptase, a mast cell marker, has demonstrated that these cells are present in 32–40% of low-grade (grade 1) and 86–90% of high-grade (grade 2 and 3) meningiomas [175,176]. However, the increased presence of mast cells in high-grade tumors has not been shown consistently in the literature; for example, Jabini et al. did not find an association between mast cells and the meningioma tumor grade [183]. Mast cells are typically located adjacent to blood vessels but may also be located more centrally and diffusely within higher grade tumors [161,175,176,184]. Notably, mast cells have been found to be associated with peritumoral edema, which can significantly contribute to the morbidity of these tumors by complicating surgery and increasing hospitalization times. High numbers of mast cells are also associated with cystic changes in meningiomas [177], and increased numbers of mast cells have been found in secretory compared to non-secretory meningiomas [185]. Furthermore, Tirokatai et al. found a potential relationship between VEGF-positive mast cells and edema in secretory meningiomas, suggesting a potential contributory role for mast cells in the development of edema [186]. Peritumoral brain edema in meningiomas has also been associated with IL-6 expression, suggesting a relationship between the immune characteristics and pathogenesis of these tumors [187].

#### 2.3.2. Structural Changes within the TME

The role of glymphatic dysfunction in meningiomas has also been investigated. The volume of peritumoral edema in meningiomas has been found to be inversely associated with the ALPS index, which serves as a measure of glymphatic function [188]. These findings correspond to findings in gliomas as well, suggesting that glymphatics play a similar role in brain tumor immunity across tumor types.

### 2.4. Other

#### 2.4.1. Chordoma

Chordomas are malignant sarcomas that occur in the skull base, vertebral bodies, or sacrum [189]. Dridi et al. conducted an immunohistochemical analysis of 81 chordomas and found that macrophages and CD4+ T cells predominated, with CD8+ T cells, CD20+ B cells, and high endothelial venules present in lesser numbers [190]. These immune infiltrates likely create an anti-inflammatory microenvironment, which is in line with the fact that M2 macrophages outnumber M1 macrophages in nearly all types of sarcomas and that these tumor cells express CD47, which downregulates proinflammatory macrophage activity [191]. Furthermore, both Mathios et al. and Dridi et al. reported that, while chordoma tumor cells do not themselves express PD-L1, this marker is expressed by infiltrating immune cells in a subset of chordomas. In these studies, PD-LI+ immune cells were associated with larger tumor size and may be negative prognostic indicators [190,192]. Yet, studies on PD-L1 expression on chordoma tumor cells have not been consistent, with Zou et al. and Feng et al. previously reporting PD-L1 expression in 66.7% and 94.9% of cases, respectively [193,194]. Further investigation is necessary to characterize the TME of these indolent tumors.

#### 2.4.2. Sellar Tumors

Lu et al. conducted a histological analysis of 35 pituitary adenomas and found that the quantity of CD68+ macrophages was positively correlated with the size and invasiveness of adenomas [195]. Further, they reported that while overall T lymphocyte infiltration in pituitary adenomas was sparse, growth-hormone-secreting adenomas had higher levels of CD4+ and CD8+ T cell infiltration than non-growth-hormone-secreting adenomas. Yagnik et al. also investigated macrophage infiltration in non-functioning pituitary tumors and found that tumors that invaded the cavernous sinus had M2/M1 macrophage ratios greater than one, whereas tumors that did not invade had M2/M1 macrophage ratios less than one [196]. This finding persisted when the authors cultured M1 and M2 macrophages with non-functioning pituitary adenoma cells and found that M2 macrophages promoted greater invasion and proliferation of adenoma cells than M1 macrophages [196].

## 3. Therapeutic Targets within the TME

The TME plays a key role in determining the malignancy and progression of CNS tumors and is relatively genetically stable compared to tumor cells [28,197]. Thus, various immunotherapeutic strategies are being considered to target the TME of CNS tumors.

### 3.1. Checkpoint Inhibitor Therapy

Given their efficacy in other cancer types, including breast, bladder, and lung cancers, immune checkpoint inhibitors have been extensively studied for use in CNS neoplasms [198]. In a murine orthotopic glioma model, combined radiation and anti-PD-1 therapy was shown to increase survival, and the survival advantage was shown to be dependent on the presence of functional CD8+ T cells [199]. Additionally, intravenously injected NK cells treated with PD-1 antibodies increased survival in a glioma mouse model, compared to treatment with NK cells alone [200]. Furthermore, Mathios et al. demonstrated that combined local chemotherapy and anti-PD-1 blockade improved survival in glioblastoma-bearing mice, compared to systemic chemotherapy or local chemotherapy alone [201].

In a small phase 1 clinical trial, Cloughesy et al. demonstrated in 35 patients that neoadjuvant therapy with PD-1 blockade for recurrent GBM was correlated with an increased progression-free and overall survival [202]. In a subsequent study using proteomics and transcriptomics to evaluate immune cell infiltration before and after neoadjuvant PD-1 blockade, it was found that the neoadjuvant PD-1 blockade increased the infiltration of CD8+ T cells but did not modify immunosuppressive macrophages [203]. In the checkmate-143 phase 3 clinical trial, using the monoclonal PD-1 antibody nivolumab, no survival advantage was demonstrated over the VEGF inhibitor bevacizumab [204]. However, post hoc subgroup analyses for this trial suggested that nivolumab may be beneficial for patients without baseline corticosteroid use and with MGMT promoter methylation [204]. Subsequent trials have separately evaluated nivolumab in patients with and without MGMT promoter methylation [205]. In the checkmate-498 phase 3 trial, nivolumab was found to have no survival advantage over temozolomide in patients without MGMT promoter methylation [205]. In the checkmate-548 trial investigating standard-of-care in combination with nivolumab or placebo in patients with newly diagnosed GBM and methylated or indeterminate MGMT promoters, no improvement in survival was demonstrated [206]. Notably, however, patients with PD-L1 expression levels greater than 5% in this study displayed a trend toward improved progression-free survival [206]. Further clinical trials investigating PD-1 blockade in specific patient populations with GBM are underway and may offer further insight into the subtypes that may benefit from this therapy (Supplementary Table S1).

One potential marker for response is a high mutational burden, which has generally been correlated with a better response to checkpoint blockade therapy in other cancers. [207]. However, understanding the cause of an increased mutational burden for each patient is critical, as different underlying mutational processes result in differing neoantigen qualities and treatment efficacies [208,209]. For example, Tuoat et al. reported no difference in survival between GBM patients with and without temozolomide-induced hypermutation who were treated with PD-1 blockade [210]. However, patients with other underlying causes of high mutational burden, such as biallelic mismatch repair deficiency and germline *POLE* deficiency, have demonstrated favorable responses to PD-1 blockade therapy [211,212]. Thus, the identification of the specific drivers of hypermutation in brain cancer patients may be necessary to elucidate the relationship between mutational burden and responsiveness to checkpoint inhibitor therapy.

CTLA-4, a glycoprotein on T cells, which blocks activation and is constitutively expressed on Tregs, also offers a potential target for immune checkpoint therapy that has been validated in other cancer types [213]. In a murine glioma model, CTLA-4 blockade was shown to enhance anti-tumor immunity via improved CD4+ T cell function and resistance to Foxp3+ Tregs [214]. However, in exploratory phase 1 cohorts of the checkmate 143 trial (investigating nivolumab with or without the CTLA-4 inhibitor ipilimumab), the combination therapy was not well-tolerated by patients [215].

Lymphocyte activation gene 3 (*LAG-3*) is another target for checkpoint inhibitor therapy. Opduoalag, which combines nivolumab (anti-PD-1) with the anti-LAG-3 drug Relatlimab, was recently approved by the FDA for use in advanced melanoma. LAG-3 expression has been demonstrated in human glioblastoma, and LAG-3 inhibition, or knockout, has been shown to have anti-GBM effects in a murine model [216]. LAG-3+ TILs are sparse in GBM and have not been shown to correlate with survival, but preclinical studies suggest that targeting LAG-3 may be a promising therapeutic strategy [216,217]. Other possible targets for checkpoint inhibitor therapy in glioblastoma include Indoleamine-pyrrole 2,3-dioxygenase (IDO), T-cell immunoglobulin mucin-3 (TIM-3), Killer-cell immunoglobulin-like receptors (KIRs), and 4-1BB (for activation rather than blockade), all of which have been investigated to varying degrees [216,218,219,220,221,222].

Notably, while in-human trials evaluating singular checkpoint-inhibitors have been thus far unable to demonstrate improvements in survival in patients with GBM, targeting more than one checkpoint and combination with other treatments may be a promising therapeutic strategy. Monotherapies with immune checkpoint inhibitors may result in the upregulation of other checkpoints, thus limiting their efficacy [204]. For example, in a murine model of lung adenocarcinoma, Koyoma et al. demonstrated that TIM-3 was upregulated after PD-1 blockade therapy, and that targeting TIM-3, following a PD-1 blockade failure, offered a survival advantage [223]. Preclinical studies in glioma models, using combination therapies, have also produced promising results [220,224]. Kim et al. evaluated a combination PD-1 and TIM-3 blockade in a murine glioma model and found that combination therapy, along with focal radiation, significantly improved long-term survival [220]. Furthermore, Belcaid et al. reported that activation of the costimulatory signal 4-1BB, in combination with CTLA-4 blockade and focal radiotherapy, improved survival in a glioma mouse model in a CD4+ T cell-dependent manner [224]. However, as in the case of combination nivolumab and ipilimumab in the checkmate-143 phase 1 cohort, combination therapies may be associated with significant toxicities [204]. Nevertheless, combination therapies may be critical in overcoming resistance, recurrence, and progression of CNS malignancies. All current clinical trials investigating checkpoint inhibitor therapy in GBM are listed in Supplementary Table S1.

While the majority of work has focused on checkpoint inhibition in gliomas, checkpoint inhibitor therapy has also been studied for potential use in other CNS neoplasms. In a meningioma mouse model, administration of the PD-1 checkpoint inhibitor avelumab along with infusion of highly active NK cells resulted in improved survival [225]. In 2017, Gelerstein et al. noted a significant reduction in the size of an intracranial meningioma in a patient being treated with the PD-1 blockade therapy nivolumab for advanced lung cancer [226]. Nidamanuri et al. performed a retrospective chart review of meningiomas in patients treated with PD-1 inhibitors at their institution and concluded that there may be a role for immune checkpoint therapy in patients with recurrent, high-grade meningiomas [227]. Currently, seven clinical trials are investigating a potential role for PD-1 or combined PD-1 and CTLA-4 blockade in recurrent or refractory meningiomas [159,227,228] (Supplementary Table S2). Phase 2 trial results from the clinical trial NCT02648997 demonstrated that, while nivolumab was well-tolerated, it did not improve six-month progression free survival [228]. Notably, however, two patients with high tumor mutational burdens were found to significantly benefit from nivolumab therapy and had substantial macrophage and lymphocytic infiltration, demonstrating a potential role for checkpoint inhibition in this patient subset [211,228].

### 3.2. Anti-Tumor Vaccines

Vaccines against brain tumors are also a promising immunotherapeutic strategy that allow for adaptive immune surveillance against cancer cells. Vaccine development for CNS neoplasms, to date, has focused on gliomas [229,230]. Peptide-based brain tumor vaccines are designed to deliver tumor specific antigens in order to activate an immune response against cancer cells [229]. One vaccine that has been extensively investigated in preclinical and clinical models is rindopepimut, which targets epidermal growth factor receptor variant III (EGFRvIII) [231]. EGFRvIII is exclusively but heterogeneously found on glioblastoma cells [229,231]. Based on success in preclinical models and improved progression free survival in three phase II clinical trials, an international phase III trial of 745 primary GBM patients was initiated to investigate combination rindopepimut and temozolomide [231]. While patients in the experimental group had a strong humoral response, no improvement in survival was noted [231]. Rindopepimut has also been investigated for recurrent GBM in combination with bevacizumab, an angiogenesis inhibitor [232]. In a randomized phase II study, combination rindopepimut and bevacizumab was found to induce regression and improve the 24-month survival rate in recurrent GBM, from 3% in controls to 20% in the treatment group, suggesting that the combination of immunotherapeutic vaccines with VEGF inhibitors may be promising [232]. Brain tumor vaccines targeting mutant IDH, which is characteristic of low-grade gliomas and has high levels of penetrance in tumor cells, have also been investigated. Schumacher et al. demonstrated the potential utility of IDH1-R132H-mutated peptide in a mouse model, in which vaccine administration induced a CD4+ T cell immune response [233]. Subsequently, the phase 1 NOA-16 and RESIST clinical trials were initiated to evaluate IDH-1R132H peptide vaccines in patients with newly diagnosed and recurrent glioma, respectively [234] (Supplementary Table S3). The majority of patients in both trials demonstrated anti-IDH1 R132H immune responses. Furthermore, in the NOA-16 trial, those who had vaccine-induced T-cell and/or B cell immune responses had a two-year progression-free survival rate of 0.82, whereas those without an immune response showed progression within two years [234]. While further results are needed to evaluate the efficacy of the vaccine in these trials, both suggest a potential therapeutic role for the IDH1-R132H vaccine in IDH-mutant gliomas.

Other peptide antigens are also being investigated for utilization in anti-glioma vaccines, including Wilms tumor 1, cytomegalovirus peptide pp6547, survivin, and telomerase reverse transcriptase [235]. Notably, given the high probability of immune escape from a single antigen, anti-tumor immunotherapeutic vaccines have also been engineered to include more than one antigen. One such vaccine is the ICT-107 vaccine, created by incubation of an autologous dendritic cell with six peptides (AIM-2, gp100, HER2, IL13Rα2, MAGE-1, and TRP-2) that are over-expressed in GBM tumor cells [236]. In a phase II study, the ICT-107 vaccine was shown to increase progression free survival by 2.2 months when compared to unpulsed dendritic cells in newly diagnosed GBM patients. Interestingly, the subset of patients with HLA-A2 antigen expression had a higher immune response via Elispot, an assay that detects functional TILs [236]. Another multipeptide vaccine, TAS0313 (including the 8 antigens EGFR, KUA, LCK, MRP3, PTHRP, SART2, SART3, and WHSC2), has also demonstrated both tolerability and immunogenicity, indicated by increased levels of IgG and cytotoxic T cells, in phase I/II studies [237]. Effector T cell responses and tolerability were also confirmed for the IMA950 multipeptide vaccine (including the 11 antigens derived from the BCAN, CSPG4, FABP7, IGF2BP3, NRCAM, NLGN4X, PTPRZ1, TNC, c-met, survivin, and HBV core antigen proteins in phase I and phase I/II trials); however, it was not shown to be effective in combination with the VEGF-inhibitor bevacizumab in a study performed by Boydell et al. [238,239,240]. Finally, the multipeptide vaccine EO2401, which features peptides derived from the microbiome that mimics tumor antigens, is currently being investigated in phase I/II trials (Supplementary Table S3).

Gliomas have also been targeted via tumor cell lysate-based vaccines, which involve the delivery of multiple immunogenic antigens from an allogeneic tumor cell lysate [241]. Ogino et al. investigated the therapeutic potential of injecting low-grade glioma patients with glioma-associated antigens in the GBM6-AD stem cell line, derived from a patient diagnosed with GBM, in order to induce an immune response and prevent the malignant transformation of low-grade gliomas [241]. They found that vaccine administration both initiated a peripheral CD8+ immune response and induced CD8+ T cell migration into the glioma microenvironment [241]. Another tumor cell-lysate based vaccine, DCVax-L, took a different approach, using autologous tumor lysate from patients’ resected glioblastoma tissue [242]. A strong T cell response was elicited by the DCVax-L in preclinical studies, and phase I trials demonstrated that patients with low TGF-β expression may be more likely to benefit from the vaccine [242]. In phase 3 trials, interim results suggested that patients were living longer, as evidenced by long tails on Kaplan–Meier survival curves (estimated median survival of 88.2 months) [243]. However, further investigation is needed to validate these findings [243]. All current clinical trials investigating immunotherapeutic vaccines in GBM are listed in Supplementary Table S3.

### 3.3. Other Therapeutic Approaches

Numerous other approaches have also been investigated as potential brain cancer therapies. Chimeric antigen receptor (CAR) T cell therapy, which involves engineering T cells to express tumor-specific chimeric antigen receptors and was approved by the Food and Drug Administration in 2017, represents a potential avenue for brain tumor therapy [244]. Notably, T cells can be designed via the introduction of costimulatory signals to have an activated phenotype, allowing them to potentially overcome the immunosuppressive brain tumor microenvironment that is characteristic of many brain tumor neoplasms [244]. As with vaccines, CAR-T cell therapy in brain cancer has largely focused on glioma [245].

To date, the targets of CAR-T cell therapy for glioblastoma include EGFRvIII, HER2, and IL13Rα2 [245]. EGFRvIII-targeting CAR-T cells have had limited success in clinical trials. In a phase I trial, the infusion of EGFRvIII-targeting CAR-T cells led to an adaptive immunosuppressive tumor response characterized by FOX3P+ Treg infiltration and the upregulation of immunosuppressive markers, including IDO-1, IL-10, PD-L1, and TGF-β [246]. In a subsequent phase I pilot trial, the administration of EGFRvIII-targeting CAR-T cells demonstrated dose-dependent adverse events, but no clinical benefit [247]. A phase I trial has also been conducted for HER-2 specific CAR-T cells. In this study, Ahmed et al. administered HER-2-specific CAR-T cells, which were constructed from virus-specific T cells in order to potentially elicit a costimulatory effect via latent virus antigens [248]. In total, eight out of seventeen patients in this trial benefitted from the treatment, achieving either a partial response or stable disease, and three patients demonstrated no progression at a 29-month follow-up [248]. CAR-T cell therapy with IL13Rα2 may also warrant further investigation, with two out of three patients in a pilot study demonstrating transient anti-glioma T-cell responses [245,249]. Other potential targets that are being investigated for use in CAR-T cell therapy in clinical studies include B7-H3, CD147, GD2, MMP2, and NKG2D, with additional targets including CAIX, CD70, CSPG4, EPhA2, and TROP2 being investigated in preclinical studies [245]. All current clinical trials investigating CAR-T cell therapy in GBM are listed in Supplementary Table S4.

CAR-T cell therapy has also been investigated in preclinical studies in other primary brain tumor types, including anaplastic meningiomas and chordomas [250,251]. Tang et al. investigated the local delivery of B7-H3-targeted CAR-T cells in a patient with recurrent anaplastic meningioma and reported that the treatment demonstrated both tolerability and local bioactivity, with decreased B7-H3 expression detected in the cavity after treatment [250]. Tumor bulk was also noted to be stable near the site of delivery for the duration of treatment [250]. B7-H3 has also been identified as a potential target for CAR-T therapy in chordoma [251]. Long et al. investigated the expression of several CAR-T targets in skull-based chordomas and found that B7-H3 was expressed in the greatest quantities. Furthermore, B7-H3-targeting CAR-T cells were demonstrated to have anti-tumor effects against in vitro tumor spheres derived from surgically resected chordoma tissue, demonstrating a potential role for CAR-T cell therapy in patients expressing this antigen [251]. Further studies are required to validate targets and evaluate the applicability of CAR-T cell therapy in brain tumor patients.

As numerous T-cell-based therapies continue to be investigated for GBM and other primary brain cancers, novel assays are needed to identify targets for T-cell anti-tumor immune responses. One such assay is the mutation-associated neoantigen functional expansion of specific T cells (MANAFEST) assay, which represents a novel strategy for identifying tumor biomarkers and predicting patients’ response to immunotherapies. Notably, this assay uses TCR sequencing and bioinformatics to identify tumor-specific TCR Vβ clonotypes and provides critical information about the anti-tumor T cell response over time, with high sensitivity and specificity [252]. This data can then be used to determine particular genomic features that are related to tumor immunogenicity and identify molecular motifs that predict patients’ innate immune responses, as well as responses to immunotherapies [252]. Other strategies to define therapeutic targets include the characterization of the tumor cell surface proteins via spatial proteomics, which can identify distinct topographic histologic patterns that are of particular interest in heterogenous tumors, such as GBM [253,254]. For example, Rose et al. utilized spatial proteomics and MALDI-mass spectrometry to identify 11 proteins that may be potential targets for the future development of cancer vaccines or CAR-T immunotherapy [253]. Advancements in antigen discovery platforms will enable the appropriate targeting of novel therapeutic modalities and further personalized brain tumor care.

As discussed, immunotherapeutic approaches against brain tumors have largely focused on restoring or engineering anti-tumor T cell responses. However, numerous other immune cell types contribute to the immunosuppressive microenvironment in brain tumors, and thus may also be viable targets. One approach being investigated is to alter the differentiation of tumor-associated macrophages (TAMs) from a pro-tumor to an anti-tumor phenotype. Specifically, preclinical studies have demonstrated that the inhibition of the colony stimulating factor 1 receptor (CSF-1), which is involved in inducing macrophage differentiation and proliferation, may be promising [255,256]. Pyonteck et al. evaluated the efficacy of CSF-1 blockade in a murine glioma model and found that CSF-1 inhibition improved survival. Specifically, the vehicle-treated cohort had a median survival of 5.7 weeks, whereas 64.3% of CSF-1-blockade-treated mice were alive at the 26-week endpoint. The CSF-1 blockade also led to tumor regression and slowed the growth of patient-derived xenografts [255]. Additionally, Stafford et al. investigated the role of CSF-1 inhibition after treatment with ionizing radiation (IR), which was shown to upregulate CSF-IR expression in GBM xenografts [256]. They found that the CSF-1R blockade prevented the differentiation of monocytes into immunosuppressive M2 polarized macrophages [256]. However, in phase II studies of the small molecule CSF-1 and KIT ligand inhibitor PLX3397, there was no improvement in the primary endpoint of 6-month progression free survival [257]. The lack of success of anti-CSF-1 monotherapy beyond preclinical models may be due to the induction of acquired resistance mechanisms, such as FOXP3+ Treg infiltration and MDSC recruitment [258,259,260]. Combining multiple therapeutic strategies may help overcome the shortcomings of monotherapy, and studies have demonstrated that the CSF-1R blockade may enhance the anti-tumoral T cell responses induced by checkpoint inhibitor therapy [259,261,262]. For example, Przystal et al. demonstrated that the CSF-1R blockade may improve the effect of anti-PD1 therapy in glioblastoma [261]. These findings support the further investigation of combinatory therapy for brain cancer.

Additionally, novel nanomedicine technologies have emerged as a potential way to modulate the immune microenvironment to slow tumor growth. For example, M1 macrophage-derived extracellular vesicles (M1EVs) delivery is another TAM-focused strategy that has been investigated recently in GBM. Wang et al. demonstrated that functionalized M1EVs were capable of penetrating the blood–brain barrier, inducing M2 to M1 polarization, and delivering and activating the chemotherapeutic agent banoxantrone [263]. Furthermore, they demonstrated that M1EVs had therapeutic effects in cell-derived and patient-derived xenografts [263]. These findings demonstrate a complementary role for nanomedicine and immunotherapeutics in the experimental treatment of glioblastoma.

Lastly, CSF-1/CSF-1R signaling has also been targeted as a therapy in high-grade meningiomas to reduce the activity of M2 phenotype immunosuppressive macrophages [264]. In a murine meningioma model, Yueng et al. demonstrated that treatment with anti-CSF-1R monoclonal antibodies, and not anti-PD-1 therapy, led to a reduction in tumor growth [264]. Thus, macrophage remodeling strategies to overcome the immunosuppressive TME may be relevant across brain tumor types.

## 4. Conclusions

The TME of CNS tumors significantly impacts the overall biology of the tumor, with important implications for patient prognosis and treatment. Research to date has allowed the sub-characterization of tumors based on TME features, as well as the development of novel therapeutic modalities targeted towards specific components of the TME. Yet, while novel therapies have produced notable results in pre-clinical studies, a majority of clinical trials to date have failed to demonstrate a significant impact on patient outcomes. However, ongoing work to advance our understanding of the complexities of the CNS TME will allow for optimized and individualized therapy selection, as well as the development of new therapeutic strategies.

## Figures and Tables

**Table 1:** Summary of immune cell infiltration of GBM.

Immune Cell Type	Relevant Literature	Characteristics
TILs	Walker et al., 2006, Zhang et al., 2019, Perng and Lim 2015, Davidson et al., 2019, Wilmotte et al., 2005, Bloch et al., 2013, Tumangelova-Yuzeir et al., 2019, DiDomenico et al., 2018, Heimberger et al., 2008, Jacobs et al., 2010, El Andaloussi et al., 2007, Lee et al., 2019, Joalland et al., 2018, Chauvin and Joalland et al., 2019, Nakazawa et al., 2014, Nakazawa et al., 2016, Bryant et al., 2009	A greater proportion of CD8+ and CD4+ T cells in GBM are apoptotic [5] Patients with GBM have limited T cell clone diversity compared to healthy individuals [6] Tumor-cell secretion of the immunosuppressive cytokines IL-10 and TGF-β suppresses cytotoxic T cell activity [7] TILs in glioma patients highly express the exhaustion marker PD-1, which may be upregulated by IL-10 [8,9,10] FOX3P+ regulatory T cells are enriched in GBM and likely contribute to the immunosuppressive environment, but their effect on survival is unclear [11,12,13,14,15] γδ T cells have anti-tumor potential but are depleted in GBM [16,17,18,19,20,21]
NK cells	Vauléon et al., 2012, Zhong et al., 2019, Bockmayr et al., 2019, Zhu et al., 2019, Kozlowska et al., 2016, Tseng et al., 2014	Activated NK cells are associated with better prognosis in glioma [22,23,24,25] Activated NK cells are effective against GBM stem cells, but less effective against differentiated GBM cells, suggesting a role for combination chemotherapy [26,27]
TAMs/Microglia	Andersen et al., 2021, Locati et al., 2020, Gregoire et al., 2020, Ransohoff et al., 2016, Friebel et al., 2020, Sankowski et al., 2019, Arrieta et al., 2021, Klemm et al., 2020, Bohn et al., 2018, Guo et al., 2016, Lin et al., 2019, Sorensen et al., 2018, Muller et al., 2017, Gabrusiewicz et al., 2016, Chen et al., 2017, Woolf et al., 2021, Gutmann et al., 2019, Hutter et al., 2019, Gholamin et al., 2017	While historically TAMs have been classified as M1 (anti-tumoral) or M2 (pro-tumoral) this classification system may be misleading [28,29,30,31] Single cell profiling studies in gliomas have revealed a variety of myeloid cell populations that go beyond classic M1/M2 phenotypes [32,33,34] Many macrophages express both M1 and M2 markers; polarization is influenced by tumor-associated factors, such as acidosis and hypoxia [35,36,37,38,39,40,41,42] Most macrophages in GBM are monocyte-derived [42] Higher ratio of microglia to bone marrow-derived macrophages is associated with increased patient survival [43] Antiphagocytic CD47 marker may impair anti-tumor phagocytic function of microglia [44,45,46]
MDSCs	Alban et al., 2020, Lakshmanachetty et al., 2021, Jia et al., 2010, Ravi and Neidert et al., 2022, Lee-Chang et al., 2019	MIF-dependent tumor cell infiltration of MDSCs is associated with poor prognosis [47] MDSCs mediate widespread immunosuppression via the release of immunosuppressive cytokines, which leads to suppression of cytotoxic T cells, NK cells, anti-inflammatory macrophages, and dendritic cells and recruitment of regulatory T and B cells and M2 macrophages [48,49,50,51]
Neutrophils	Fridlender et al., 2009, Lin et al., 2021, Magod et al., 2021, Andzinski et al., 2016, Scapini et al., 2000, Sionov et al., 2015, Yee et al., 2020	Neutrophils were previously thought to be N1 or N2 polarized, but recent studies show they span a wide spectrum of activation states [52,53] During tumor progression, neutrophil function transitions from anti-tumor to pro-tumor [54] Proinflammatory neutrophils release cytokines and chemokines (IL-12, VEGF, TNF-α, GM-CSF, CCL3, CXCL9, and CXCL10) to recruit cytotoxic cells [55,56,57] Neutrophil-mediated ferroptosis leads to GBM tumor necrosis and is correlated with worse patient outcomes [58]
Dendritic cells	Kastenmüller et al., 2013, Srivastava et al., 2019, Mikucki et al., 2015, Wendel et al., 2008, Mittal et al., 2017, Hochrein et al., 2000, Zong et al., 2016	Type 1 polarized dendritic cells induce proinflammatory TH1 responses and have anti-tumor effects [59,60,61,62,63,64] The immunosuppressive glioma microenvironment may induce a pro-tumor regulatory dendritic cell phenotype via expression of VEGF, PGE2, and IL-10 [65]
CAFs	Li et al., 2020, Kim et al., 2022, Chen et al., 2021, Trylcova et al., 2015	Fibroblast activating proteins including FAPα and PDGFRβ are expressed in GBM, and small quantities of fibroblasts have been identified in resected human GBM samples [66,67] Expression of CAF-related genes is associated with higher risk in gliomas and may influence chemotactic migration of glioma cells [68,69]

**Table 2:** Key differences in the immune cell infiltration of high- and low-grade gliomas.

Immune Cell Type	Relevant Literature	Characteristics in High- vs. Low-Grade Glioma
TILs	Haddad et al., 2022, Klemm et al., 2020, Weenink et al., 2019, Kohanbash et al., 2017, Berghoff et al., 2015, Berghoff et al., 2016, Garber et al., 2016, Heimberger et al., 2008, Friebel et al., 2020	Low-grade gliomas have lower numbers of TILs [35,145] Decreased expression of T-cell trafficking CXCL9 and CXCL10 in low-grade compared to high-grade gliomas [147,148] Lower levels of PD-1-expressing TILs in low-grade gliomas, suggesting a less exhausted phenotype compared to high-grade [149,150,151] Lower levels of T regulatory cells in low-grade compared to high-grade gliomas [14,32]
NK cells	Ren et al., 2019, Huang et al., 2006	There is increased NK cell infiltration in low-grade compared to high-grade tumors and NK cell infiltration is associated with better prognosis [152] Expression of CXCL13, which plays a role in NK cell recruitment, is also increased in lower grade gliomas [152,153]
TAMs/microglia	Vidyarthi et al., 2019, Venteicher et al., 2017	Fewer M2 and greater M1 macrophages in low- vs. high-grade gliomas [154] Tumor progression to higher grades is associated with a greater macrophage to microglia ratio [155]

**Table 3:** Summary of immune cell infiltration of meningiomas.

Immune Cell Type	Relevant Literature	Characteristics
TILs	Rapp et al., 2019, Berghoff et al., 2020, Fang et al., 2013, Zador et al., 2020	High levels of TILs are associated with improved progression free survival in high-grade meningiomas [162] FOXP3+ regulatory T cells are enriched in high-grade meningiomas [163] PD-L1-expressing tumor cells are increased in proportion to meningioma tumor grade [164] Recurrent meningiomas are characterized by lower numbers of TILs [162] Skull base meningiomas exhibit higher levels of γδ T cell infiltration [165]
TAMs/microglia	Borch et al., 2021, Proctor et al., 2019, Asai et al., 1999, Morantz et al., 1979, Domingues at al. 2013, Presta et al., 2017, Grund et al., 2009, Woolf et al., 2021	TAMs constitute 18–44% of meningioma immune infiltrate [157,166,167,168,169] The M2 phenotype predominates in meningiomas and is positively associated with tumor size, grade, and recurrence [166,170] In total, 25% of meningiomas had macrophagic or microglial cells at the tumor border, suggestive of invasive tumor behavior [171] Meningiomas have low levels of microglia [43]
MDSCs	Li et al., 2019, Pinton et al., 2018	High-grade meningiomas have greater MDSC infiltration than low-grade meningiomas [172] Infiltrating MDSCs in high-grade meningiomas reduce T cell activity [173]
Dendritic cells	Chen et al., 2020	In meningiomas, lower levels of dendritic cells are associated with survival, likely via a reduction in B-cell receptor signaling [174]
Mast cells	Reszec et al., 2012, Reszec et al., 2013, Schober et al., 1988	Mast cells are present in 32–40% of low-grade and 86–90% of high-grade meningiomas [175,176] The development of peritumoral edema is associated with the presence of mast cells [176] High numbers of mast cells may lead to cystic changes in meningiomas [177]

## Supplementary Materials

The following supporting information can be downloaded at: https://www.mdpi.com/article/10.3390/ijms24032020/s1.
