# Supplementary material for: The Tumor Immune Microenvironment in Primary CNS Neoplasms: A Review of Current Knowledge and Therapeutic Approaches
Source: Int J Mol Sci. 2023 Jan 19;24(3):2020. doi: 10.3390/ijms24032020 (PMC9917056; doi:10.3390/ijms24032020)
Supplement: Supplementary file 1 [file ijms-24-02020-s001.zip › ijms-2058144-supplementary.pdf]

Supplementary Table S1| Current checkpoint inhibitor clinical trials for glioma that are active, recruiting, enrolling by invitation, or not yet recruiting

NCT Number	Title	Status	Conditions	Interventions	Phase	Enrollment	Start Date
NCT02658279	Pembrolizumab (MK-3475) in Patients with Recurrent Malignant Glioma with a Hypermutator Phenotype	Active, not recruiting	Glioma;<br>Recurrent Malignant Glioma	Pembrolizumab	Not Applicable	27	22-Jan-16
NCT02829931	Hypofractionated Stereotactic Irradiation with Nivolumab, Ipilimumab and Bevacizumab in Patients with Recurrent High-Grade Gliomas	Active, not recruiting	Malignant Glioma	Hypofractionated Stereotactic Radiation<br>Nivolumab<br>Bevacizumab<br>Ipilimumab	Phase 1	33	22-Aug-16
NCT02658981	Anti-LAG-3 Alone & in Combination w/ Nivolumab Treating Patients w/ Recurrent GBM (Anti-CD137 Arm Closed 10/16/18)	Active, not recruiting	Glioblastoma;<br>Gliosarcoma;<br>Recurrent Brain Neoplasm	Anti-LAG-3 Monoclonal Antibody BMS 986016<br>Anti-CD137<br>Nivolumab	Phase 1	63	24-Aug-16
NCT03233152	Intra-tumoral Ipilimumab Plus Intravenous Nivolumab Following the Resection of Recurrent Glioblastoma	Recruiting	Glioblastoma	Ipilimumab<br>Nivolumab	Phase 1	6	17-Nov-16
NCT03425292	A Longitudinal Assessment of Tumor Evolution in Patients with Brain Cancer	Recruiting	Newly Diagnosed High Grade Glioma	Temozolomide<br>conformal brain radiation therapy<br>Nivolumab<br>Ipilimumab<br>Bevacizumab<br>Lomustine	Phase 1	90	1-Mar-18
NCT03426891	Pembrolizumab and Vorinostat Combined with Temozolomide for Newly Diagnosed Glioblastoma	Active, not recruiting	Glioblastoma;<br>Brain Tumor;<br>GBM	Pembrolizumab<br>Vorinosta<br>Temozolomide<br>Radiotherapy	Phase 1	21	16-Mar-18
NCT04047706	Nivolumab, BMS-986205, and Radiation Therapy with or without Temozolomide in Treating Patients with Newly Diagnosed Glioblastoma	Recruiting	Glioblastoma	IDO1 Inhibitor BMS-986205<br>Nivolumab| Radiation Therapy<br>Temozolomide	Phase 1	30	13-Aug-19
NCT04201873	Pembrolizumab and a Vaccine (ATL-DC) for the Treatment of Surgically Accessible Recurrent Glioblastoma	Recruiting	Recurrent Glioblastoma	Dendritic Cell Tumor Cell Lysate Vaccine<br>Pembrolizumab<br>Poly ICLC	Phase 1	40	8-Jan-20
NCT04160494	D2C7-IT With Atezolizumab for Recurrent Gliomas	Recruiting	Malignant Glioma	D2C7-IT<br>Atezolizumab	Phase 1	18	20-Mar-20
NCT04521686	Study of LY3410738 Administered to Patients with Advanced Solid Tumors with IDH1 or IDH2 Mutations	Recruiting	Cholangiocarcinoma;<br>Chondrosarcoma;<br>Glioma;<br>Any Solid Tumor	LY3410738<br>Gemcitabine<br>Cisplatin<br>Durvalumab	Phase 1	200	20-Aug-20
NCT04606316	Surgical Nivolumab and Ipilimumab for Recurrent GBM	Recruiting	Glioblastoma;<br>GBM;	Nivolumab<br>Ipilimumab<br>Surgery	Phase 1	60	1-Feb-21

			Glioblastoma Multiforme; Grade IV Astrocytoma				
NCT04826393	ASP8374 + Cemiplimab in Recurrent Glioma	Active, not recruiting	Glioblastoma; Recurrent Glioblastoma	ASP8374<br>Cemiplimab	Phase 1	14	9-Mar-22
NCT03277638	Laser Interstitial Thermotherapy (LITT) Combined with Checkpoint Inhibitor for Recurrent GBM (RGBM)	Recruiting	Glioblastoma, Adult	Pembrolizumab<br>Laser Interstitial Thermotherapy	Phase 1|2	34	29-Nov-17
NCT05053880	A Study to Evaluate Safety and Efficacy of ACT001 and Anti-PD-1 in Patients with Surgically Accessible Recurrent Glioblastoma Multiforme	Recruiting	Recurrent Glioblastoma Multiforme (GBM)	ACT001<br>Pembrolizumab	Phase 1|2	48	22-Sep-21
NCT04977375	Trial of Anti-PD-1 Immunotherapy and Stereotactic Radiation in Patients with Recurrent Glioblastoma	Recruiting	Glioblastoma Multiforme	Pembrolizumab<br>Stereotactic Radiation Therapy<br>Surgical Resection	Phase 1|2	30	9-Dec-21
NCT05084430	Study of Pembrolizumab and M032 (NSC 733972)	Active, not recruiting	Glioblastoma Multiforme; Anaplastic Astrocytoma; Gliosarcoma	M032<br>Pembrolizumab	Phase 1|2	28	25-Feb-22
NCT02337686	Pembrolizumab in Treating Patients with Recurrent Glioblastoma	Active, not recruiting	Recurrent Glioblastoma; Recurrent Gliosarcoma	Pembrolizumab<br>Conventional Surgery	Phase 2	20	28-Apr-15
NCT03018288	Radiation Therapy Plus Temozolomide and Pembrolizumab with and without HSPPC-96 in Newly Diagnosed Glioblastoma (GBM)	Active, not recruiting	Glioblastoma	Pembrolizumab<br>HSPPC-96<br>Temozolomide	Phase 2	90	21-Sep-17
NCT03452579	Nivolumab Plus Standard Dose Bevacizumab Versus Nivolumab Plus Low Dose Bevacizumab in GBM	Active, not recruiting	Glioblastoma	Nivolumab<br>Low Dose Bevacizumab<br>Standard Dose Bevacizumab	Phase 2	90	21-May-18
NCT03557359	Nivolumab for Recurrent or Progressive IDH Mutant Gliomas	Active, not recruiting	Gliomas	Nivolumab	Phase 2	20	12-Jun-18
NCT03661723	Pembrolizumab and Reirradiation in Bevacizumab Naïve and Bevacizumab Resistant Recurrent Glioblastoma	Active, not recruiting	Glioblastoma	Pembrolizumab<br>Bevacizumab<br>Re-irradiation	Phase 2	61	28-Sep-18
NCT04056910	Ivosidenib (AG-120) with Nivolumab in IDH1 Mutant Tumors	Recruiting	Advanced Solid Tumor; IDH1 Mutation Glioma	Ivosidenib<br>Nivolumab	Phase 2	35	14-Aug-19
NCT03718767	Nivolumab in Patients with IDH-Mutant Gliomas with and without Hypermutator Phenotype	Recruiting	Glioma; Glioblastoma; High Grage Glioma; Low Grade Glioma;	Nivolumab	Phase 2	95	27-Mar-19

			Malignant Glioma				
NCT03743662	Nivolumab with Radiation Therapy and Bevacizumab for Recurrent MGMT Methylated Glioblastoma	Active, not recruiting	Glioblastoma	Re-irradiation<br>Bevacizumab<br>Nivolumab<br>Re-resection	Phase 2	39	12-Nov-18
NCT03899857	Pembrolizumab for Newly Diagnosed Glioblastoma	Active, not recruiting	Newly Diagnosed Glioblastoma	Pembrolizumab	Phase 2	56	21-Oct-20
NCT04013672	Study of Pembrolizumab Plus SurVaxM for Glioblastoma at First Recurrence	Active, not recruiting	Recurrent Glioblastoma	Pembrolizumab<br>SurVaxM<br>Sargramostim<br>Montanide ISA 51	Phase 2	40	19-Mar-20
NCT04145115	A Study Testing the Effect of Immunotherapy (Ipilimumab and Nivolumab) in Patients with Recurrent Glioma with Elevated Mutational Burden	Recruiting	Diffuse Glioma;<br>Recurrent Astrocytoma, IDH-Mutant, Grade 4;<br>Recurrent Glioblastoma, IDH-Wildtype;<br>Secondary Glioblastoma	Ipilimumab<br>Nivolumab	Phase 2	37	30-Oct-20
NCT04195139	Nivolumab and Temozolomide Versus Temozolomide Alone in Newly Diagnosed Elderly Patients With GBM	Active, not recruiting	Glioblastoma Multiforme	Nivolumab<br>Temozolomide	Phase 2	103	22-Feb-18
NCT04479241	LUMINOS-101: Lerapolturev (PVSRIPO) and Pembrolizumab in Patients with Recurrent Glioblastoma	Active, not recruiting	Glioblastoma;<br>Recurrent Glioblastoma;<br>Supratentorial Glioblastoma;<br>Brain Tumor	Lerapolturev<br>pembrolizumab	Phase 2	30	21-Oct-20
NCT04583020	Neoadjuvant PD-1 in Newly Diagnosed Glioblastoma	Recruiting	Glioblastoma	Camrelizumab<br>Radiation<br>Temozolomide	Phase 2	42	12-Nov-20
NCT04588987	Neoadjuvant Carilizumab and Apatinib for Recurrent High-Grade Glioma	Recruiting	Neoadjuvant Therapy;<br>Carilizumab;<br>Apatinib;<br>Recurrent High-Grade Glioma	Biological: PD-1	Phase 2	60	Oct-20
NCT04729959	Testing the Addition of the Immune Therapy Drugs, Tocilizumab and Atezolizumab, to Radiation Therapy for Recurrent Glioblastoma	Recruiting	Diffuse Astrocytoma, IDH-Wildtype;<br>Recurrent Glioblastoma	Atezolizumab<br>Conventional Surgery<br>Fractionated Stereotactic Radiation Therapy<br>Tocilizumab	Phase 2	12	6-Jul-21
NCT04817254	Association of Peripheral Blood Immunologic Response to Therapeutic Response to Adjuvant Treatment with Immune Checkpoint Inhibition (ICI) in Patients with	Recruiting	Glioblastoma;<br>Gliosarcoma;<br>Malignant Glioma	TMZ<br>Ipilimumab<br>Nivolumab	Phase 2	48	8-Dec-21

	Newly Diagnosed Glioblastoma or Gliosarcoma						
NCT04952571	Combined Treatment of Camrelizumab and Bevacizumab for Adult Patients with Recurrent Glioblastoma (GBM)	Recruiting	Recurrent Glioblastoma	Camrelizumab<br>Bevacizumab	Phase 2	94	1-May-21
NCT05074992	A Trial of Neoadjuvant Therapy in Patients with Newly Diagnosed Glioblastoma	Recruiting	Glioblastoma	Drug: Ipilimumab	Phase 2	43	24-Aug-22
NCT05366062	ERC1671 to Treat Malignant Gliomas When Given in Combination With GM-CSF, Cyclophosphamide, Bevacizumab and Pembrolizumab	Not yet recruiting	Glioma, Malignant	ERC1671<br>GM-CSF<br>Cyclophosphamide<br>Pembrolizumab	Phase 2	28	1-Jul-22
NCT05465954	Efineptakin Alfa and Pembrolizumab for the Treatment of Recurrent Glioblastoma	Not yet recruiting	High Grade Astrocytic Tumor; Recurrent Glioblastoma, IDH-Wildtype; Recurrent Gliosarcoma	Efineptakin alfa<br>Pembrolizumab	Phase 2	34	15-Nov-22
NCT02017717	A Study of the Effectiveness and Safety of Nivolumab Compared to Bevacizumab and of Nivolumab with or Without Ipilimumab in Glioblastoma Patients	Active, not recruiting	Recurrent Glioblastoma	Nivolumab<br>Bevacizumab<br>Ipilimumab	Phase 3	529	7-Feb-14
NCT02667587	An Investigational Immunotherapy Study of Temozolomide Plus Radiation Therapy with Nivolumab or Placebo, for Newly Diagnosed Patients with Glioblastoma (GBM, a Malignant Brain Cancer)	Active, not recruiting	Brain Neoplasms	Nivolumab<br>Temozolomide<br>Radiotherapy	Phase 3	716	9-May-16
NCT05235737	The Assessment of Immune Response in Newly Diagnosed Glioblastoma Patients Treated with Pembrolizumab	Not yet recruiting	Newly Diagnosed Glioblastoma	Pembrolizumab	Phase 4	36	1-Mar-22

Results obtained from clinicaltrials.gov by searching entries with Glioma OR GBM OR Glioblastoma and selecting relevant studies investigating checkpoint inhibitors (excluding missing info, unknown status, and pediatric tumors)

Supplementary Table S2| Current checkpoint inhibitor clinical trials for meningioma that are active, recruiting, enrolling by invitation, or not yet recruiting

NCT Number	Title	Status	Conditions	Interventions	Phases	Enrollment	Start Date
NCT04728568	Exploratory Study of PD-1 Neoadjuvant Treatment of Recurrent Meningioma	Recruiting	Meningioma, Malignant	Sintilimab	Not Applicable	15	1-Jun-20
NCT03267836	Neoadjuvant Avelumab and Hypofractionated Proton Radiation Therapy Followed by Surgery for Recurrent Radiation-refractory Meningioma	Active, not recruiting	Meningioma; Meningioma, Adult	Avelumab<br>Proton Therapy<br>Surgery	Phase 1	9	10-Jan-18
NCT03604978	Nivolumab and Multi-fraction Stereotactic Radiosurgery with or Without Ipilimumab in Treating Patients with Recurrent Grade II-III Meningioma	Recruiting	Grade 2 Meningioma; Grade 3 Meningioma; Recurrent Meningioma	Ipilimumab<br>Nivolumab<br>Stereotactic Radiosurgery	Phase 1|Phase 2	15	21-Nov-18
NCT02648997	An Open-Label Phase II Study of Nivolumab in Adult Participants with Recurrent High-Grade Meningioma	Recruiting	Meningiomas	Nivolumab - 240 mg<br>Ipilimumab - 1 mg/kg<br>Nivolumab - 480 mg<br>Nivolumab - 3 mg/kg| External Beam RT	Phase 2	50	Mar-16
NCT03173950	Immune Checkpoint Inhibitor Nivolumab in People with Recurrent Select Rare CNS Cancers	Recruiting	Medulloblastoma;<br>Ependymoma;<br>Pineal Region Tumors;<br>Choroid Plexus Tumors;<br>Atypical/Malignant Meningioma	Nivolumab	Phase 2	180	13-Jul-17
NCT03279692	Phase II Trial of Pembrolizumab in Recurrent or Residual High-Grade Meningioma	Active, not recruiting	High Grade Meningioma	Pembrolizumab	Phase 2	26	7-Nov-17
NCT04659811	Stereotactic Radiosurgery and Immunotherapy (Pembrolizumab) for the Treatment of Recurrent Meningioma	Recruiting	Grade I Meningioma, Adult;<br>Grade II Meningioma, Adult;<br>Grade III Meningioma, Adult;<br>Recurrent Meningioma	Pembrolizumab<br>Stereotactic Radiosurgery	Phase 2	90	25-Mar-21

Results obtained from clinicaltrials.gov by searching entries with meningioma and selecting relevant studies investigating checkpoint inhibitors (excluding missing info, unknown status, and pediatric tumors)

Supplementary Table S3| Current vaccine clinical trials for glioma that are active, recruiting, enrolling by invitation, or not yet recruiting

NCT Number	Title	Status	Conditions	Interventions	Phases	Enrollment	Start Date
NCT05557240	Neoantigens Phase I Trial in Newly Diagnosed Glioblastoma Patients	Recruiting	Glioma, Malignant|	NeoPep Vaccine1 plus Poly-ICLC<br>NeoPep Vaccine2 plus Poly-ICLC	Not Applicable	10	13-Sep-22
NCT01957956	Vaccine Therapy and Temozolomide in Treating Patients with Newly Diagnosed Glioblastoma	Active, not recruiting	Giant Cell Glioblastoma;<br>Glioblastoma;<br>Gliosarcoma	Malignant Glioma Tumor Lysate-Pulsed Autologous Dendritic Cell Vaccine<br>Temozolomide	Early Phase 1	21	11-Nov-13
NCT03360708	Vaccine Therapy in Treating Patients with Recurrent Glioblastoma	Active, not recruiting	Giant Cell Glioblastoma;<br>Recurrent Glioblastoma;<br>Recurrent Gliosarcoma	Malignant Glioma Tumor Lysate-Pulsed Autologous Dendritic Cell Vaccine	Early Phase 1	20	3-Jun-19
NCT00639639	Vaccine Therapy in Treating Patients with Newly Diagnosed Glioblastoma Multiforme	Active, not recruiting	Malignant Neoplasms of Brain	Tetanus toxoid<br>Therapeutic autologous dendritic cells<br>Therapeutic autologous lymphocytes	Phase 1	42	Jan-06
NCT02287428	Personalized NeoAntigen Cancer Vaccine w RT Plus Pembrolizumab for Patients with Newly Diagnosed GBM	Recruiting	Glioblastoma	Radiation Therapy<br>Personalized NeoAntigen Vaccine<br>Pembrolizumab<br>Temozolomide	Phase 1	56	Nov-14
NCT02193347	IDH1 Peptide Vaccine for Recurrent Grade II Glioma (RESIST)	Active, not recruiting	Brain Cancer;<br>Brain Neoplasm, Primary;<br>Brain Neoplasms, Recurrent;<br>Brain Tumor<br>Cancer of the Brain	PEPIDH1M vaccine<br>Tetanus-Diphtheria Toxoid (Td)<br>Temozolomide	Phase 1	24	28-Jan-16
NCT02549833	Neo-adjuvant Evaluation of Glioma Lysate Vaccines in WHO Grade II Glioma	Active, not recruiting	Oligodendroglioma;<br>Astrocytoma, Grade II;<br>Glioma, Astrocytic;<br>Glioma; Malignant;<br>Glioma;<br>Oligoastrocytoma, Mixed	GBM6-AD and poly-ICLC before and after surgery<br>GBM6-AD and poly-ICLC after surgery only	Phase 1	28	17-Oct-16
NCT02924038	A Study of Varlilumab and IMA950 Vaccine Plus Poly-ICLC in Patients with WHO Grade II Low-Grade Glioma (LGG)	Active, not recruiting	Glioma;<br>Malignant Glioma;<br>Astrocytoma, Grade II;<br>Oligodendroglioma;<br>Glioma, Astrocytic;<br>Oligoastrocytoma, Mixed	IMA950<br>poly-ICLC<br>Varlilumab	Phase 1	14	3-Apr-2017
NCT03893903	AMPLIFYing NEOepitope-specific VACcine Responses in Progressive Diffuse Glioma	Recruiting	Malignant Glioma	IDH1R132H peptide vaccine<br>Avelumab	Phase 1	60	19-Oct-18

NCT03914768	Immune Modulatory DC Vaccine Against Brain Tumor	Enrolling by invitation	Diffuse Intrinsic Pontine Glioma; Glioblastoma	Immunomodulatory DC vaccine to target DIPG and GBM	Phase 1	10	31-Mar-19
NCT04201873	Pembrolizumab and a Vaccine (ATL-DC) for the Treatment of Surgically Accessible Recurrent Glioblastoma	Recruiting	Recurrent Glioblastoma	Dendritic Cell Tumor Cell Lysate Vaccine<br>Pembrolizumab<br>Poly ICLC	Phase 1	40	8-Jan-20
NCT04642937	Study of CD200 Activation Receptor Ligand (CD200AR-L) and Allogeneic Tumor Lysate Vaccine Immunotherapy for Recurrent Glioblastoma	Active, not recruiting	Glioblastoma	hP1A8	Phase 1	24	1-Dec-20
NCT04842513	Multi Peptide Vaccination with XS15 in Addition to Standard Postoperative Radiation Therapy and Temozolomide Chemotherapy in Newly Diagnosed Glioblastoma	Recruiting	Glioblastoma Multiforme of Brain	Multipeptide plus XS15	Phase 1	15	3-May-21
NCT04943718	Personalized Vaccine for Patients with Recurrent Malignant Glioma	Recruiting	Malignant Glioma; Recurrent Glioma	Personalized vaccine	Phase 1	10	15-Jul-21
NCT04968366	Safety & Efficacy of DC Vaccine and TMZ for the Treatment of Newly diagnosed Glioblastoma After Surgery	Recruiting	Glioblastoma Multiforme of Brain	Autologous dendritic cells pulsed with multiple neoantigen peptides<br>Temozolomide adjuvant chemotherapy	Phase 1	10	30-Jul-21
NCT04552886	Dendritic Cell Vaccination with Standard Postoperative Chemoradiation for the Treatment of Adult Glioblastoma	Recruiting	Glioblastoma	TH-1 Dendritic Cell Immunotherapy	Phase 1	24	11-Oct-21
NCT04963413	RENEW: Feasibility of CMV RNA-Pulsed Dendritic Cells Vaccines for the Treatment of Newly Diagnosed Glioblastoma Patients.	Recruiting	Glioblastoma	Autologous DCs derived from PBMC loaded with RNA encoding the human CMV matrix protein pp65-flLAMP plus GM-CSF	Phase 1	10	13-Jan-22
NCT05283109	ETAPA I: Peptide-based Tumor Associated Antigen Vaccine in GBM	Not yet recruiting	Glioma, Malignant	Tumor Associated Antigen Peptide Vaccine<br>P30-EPS Vaccine<br>Hiltonol	Phase 1	36	Nov-22
NCT04801147	Immunotherapy With Autologous Tumor Lysate-Loaded Dendritic Cells In Patients with Newly Diagnosed Glioblastoma Multiforme	Recruiting	Glioblastoma	Biological: Dendritic Cells Vaccine	Phase 1|Phase 2	76	Jun-10
NCT02649582	Adjuvant Dendritic Cell-immunotherapy Plus Temozolomide in Glioblastoma Patients	Recruiting	Glioblastoma Multiforme of Brain	Dendritic cell vaccine plus temozolomide chemotherapy	Phase 1|Phase 2	20	Dec-15
NCT03382977	Study to Evaluate Safety, Tolerability, and Optimal Dose of Candidate GBM Vaccine VBI-1901 in Recurrent GBM Subjects	Active, not recruiting	Glioblastoma Multiforme	VBI-190<br>Carmustine<br>Lomustine	Phase 1|Phase 2	98	6-Dec-17
NCT04116658	First-in-Human, Phase 1b/2a Trial of a Multipeptide Therapeutic Vaccine	Recruiting	Glioblastoma, Adult	Multiple doses of EO2401	Phase 1|Phase 2	52	13-Jul-20

	in Patients with Progressive Glioblastoma							
NCT04388033	Clinical Study of an Dendritic and Glioma Cells Fusion Vaccine With IL-12 for Treatment-naïve GBM Patients.	Recruiting	Glioblastoma; Glioma; Neoplasms, Neuroepithelial; Neuroectodermal Tumors; Neoplasms, Nerve Tissue	Dendritic Cell<br>Tumor Fusion Vaccine<br>Interleukin-12<br>Temozolomide	Phase 1|Phase 2	10	Dec-20
NCT01204684	Dendritic Cell Vaccine for Patients with Brain Tumors	Active, not recruiting	Glioma; Anaplastic Astrocytoma; Anaplastic Astro-oligodendroglioma; Glioblastoma	Autologous tumor lysate-pulsed DC vaccination<br>Tumor lysate-pulsed DC vaccination+0.2% resiquimod<br>Tumor-lysate pulsed DC vaccination +adjuvant polyICLC	Phase 2	60	8-Oct-10
NCT01814813	Vaccine Therapy with Bevacizumab Versus Bevacizumab Alone in Treating Patients with Recurrent Glioblastoma Multiforme That Can Be Removed by Surgery	Active, not recruiting	Recurrent Glioblastoma; Recurrent Adult Brain Tumor; Gliosarcoma	HSPPC-96<br>Bevacizumab	Phase 2	90	May-13
NCT02358187	A Vaccine Trial for Low Grade Gliomas	Recruiting	Low Grade Glioma	HLA-A2 Restricted Glioma Antigen-Peptides with Poly-ICLC	Phase 2	25	Jan-15
NCT02455557	SurVaxM Vaccine Therapy and Temozolomide in Treating Patients with Newly Diagnosed Glioblastoma	Active, not recruiting	Glioblastoma; Gliosarcoma	Montanide ISA 51 VG<br>Sargramostim<br>SVN53-67/M57-KLH Peptide Vaccine<br>Temozolomide	Phase 2	66	4-May-15
NCT02465268	Vaccine Therapy for the Treatment of Newly Diagnosed Glioblastoma Multiforme	Recruiting	Glioblastoma Multiforme; Glioblastoma; Malignant Glioma Astrocytoma, Grade IV; GBM	pp65-shLAMP DC with GM-CSF<br>unpulsed PBMC and saline<br>pp65-flLAMP DC with GM-CSF	Phase 2	175	Aug-16
NCT03395587	Efficiency of Vaccination with Lysate-loaded Dendritic Cells in Patients with Newly Diagnosed Glioblastoma	Recruiting	Glioblastoma	Autologous, tumor lysate-loaded, mature dendritic cells (DC)<br>standard therapy	Phase 2	136	6-Mar-18
NCT03400917	Autologous Dendritic Cells Loaded with Autologous Tumor Associated Antigens for Treatment of Newly Diagnosed Glioblastoma	Active, not recruiting	Newly Diagnosed Glioblastoma	Biological: AV-GBM-1	Phase 2	55	20-Jun-18
NCT03688178	DC Migration Study to Evaluate TReg Depletion in GBM Patients with and Without Varlilumab	Recruiting	Glioblastoma	Human CMV pp65-LAMP mRNA-pulsed autologous DCs<br>Temozolomide Varlilumab<br>Unpulsed DCs	Phase 2	80	26-Aug-20
NCT04523688	Vaccination With Autologous Dendritic Cells Loaded with	Not yet recruiting	Glioblastoma	Autologous Dendritic Cells (DC) vaccine	Phase 2	28	Mar-21

	Autologous Tumour Homogenate in Glioblastoma			Temozolomide			
NCT04888611	Neoadjuvant PD-1 Antibody Alone or Combined with DC Vaccines for Recurrent Glioblastoma	Recruiting	Recurrent Glioblastoma	Camrelizumab plus GSC-DCV<br>Camrelizumab plus Placebo	Phase 2	40	26-Oct-21
NCT05163080	SurVaxM Plus Adjuvant Temozolomide for Newly Diagnosed Glioblastoma (SURVIVE)	Recruiting	Newly Diagnosed Glioblastoma	SurVaxM	Phase 2	265	18-Nov-21
NCT03548571	Dendritic Cell Immunotherapy Against Cancer Stem Cells in Glioblastoma Patients Receiving Standard Therapy	Active, not recruiting	Glioblastoma	Dendritic cell immunization<br>Adjuvant temozolomide	Phase 2|Phase 3	60	26-Apr-18
NCT00045968	Study of a Drug [DCVax®-L] to Treat Newly Diagnosed GBM Brain Cancer	Active, not recruiting	Glioblastoma Multiforme;<br>Glioblastoma;<br>GBM;<br>Grade IV Astrocytoma;<br>Glioma;<br>Brain Cancer;<br>Brain Tumor	Dendritic cell immunotherapy	Phase 3	348	Dec-06
NCT04277221	ADCTA for Adjuvant Immunotherapy in Standard Treatment of Recurrent Glioblastoma Multiforme (GBM)	Recruiting	Glioblastoma Multiforme	Autologous Dendritic Cell/Tumor Antigen, ADCTA	Phase 3	118	19-Sep-19
NCT05100641	AV-GBM-1 vs Control as Adjunctive Therapy After RT/TMZ for Newly Diagnosed GBM	Not yet recruiting	Primary Glioblastoma	AV-GBM-Autologous monocytes	Phase 3	726	Mar-22

Results obtained from clinicaltrials.gov by searching entries with Glioma OR GBM OR Glioblastoma AND vaccine and selecting relevant studies (Excluding missing info, unknown status, and pediatric tumors)

Supplementary Table S4| Current CAR-T cell clinical trials in glioma that are active, recruiting, enrolling by invitation, or not yet recruiting

NCT Number	Title	Status	Conditions	Interventions	Phases	Enrollment	Start Date
NCT04717999	Pilot Study of NKG2D CAR-T in Treating Patients with Recurrent Glioblastoma	Not yet recruiting	Recurrent Glioblastoma	NKG2D CAR-T	Not Applicable	20	1-Sep-21
NCT05024175	Long-term Follow-up of Subjects Treated with CARv3-TEAM-E T Cells	Not yet recruiting	Glioblastoma Recurrent, EGFR vIII Mutant;<br>Newly Diagnosed Glioblastoma, EGFRvIII Mutant;<br>Recurrent Glioblastoma, EGFR vIII Negative	CARv3-TEAM-E T cells	Not Applicable	18	Dec-21
NCT02208362	Genetically Modified T-cells in Treating Patients with Recurrent or Refractory Malignant Glioma	Active, not recruiting	Recurrent Glioblastoma;<br>Recurrent Malignant Glioma;<br>Recurrent WHO Grade II Glioma;<br>Recurrent WHO Grade III Glioma;<br>Refractory Glioblastoma;<br>Refractory Malignant Glioma;<br>Refractory WHO Grade II Glioma;<br>Refractory WHO Grade III Glioma	IL13Ralpha2-specific Hinge-optimized 4-1BB-co-stimulatory CAR/Truncated CD19-expressing Autologous TN/MEM Cells<br><br>IL13Ralpha2-specific Hinge-optimized 41BB-co-stimulatory CAR Truncated CD19-expressing Autologous T-Lymphocytes	Phase 1	82	18-May-15
NCT03423992	Personalized Chimeric Antigen Receptor T Cell Immunotherapy for Patients with Recurrent Malignant Gliomas	Recruiting	Glioma;<br>Malignant Glioma of Brain	Chimeric antigen receptor T cells	Phase 1	100	2-Mar-18
NCT03389230	Memory-Enriched T Cells in Treating Patients with Recurrent or Refractory Grade III-IV Glioma	Recruiting	Glioblastoma;<br>Malignant Glioma;<br>Recurrent Glioma;<br>Refractory Glioma;<br>WHO Grade III Glioma	HER2(EQ)BBζ/CD19t+ T cells<br>Leukapheresis	Phase 1	42	14-Aug-18
NCT03170141	Immunogene-modified T (IgT) Cells Against Glioblastoma Multiforme	Enrolling by invitation	Glioblastoma Multiforme of Brain;<br>Glioblastoma Multiforme	Antigen-specific IgT cells	Phase 1	20	31-May-19

NCT04003649	IL13Ra2-CAR T Cells with or without Nivolumab and Ipilimumab in Treating Patients With GBM	Recruiting	Recurrent Glioblastoma; Refractory Glioblastoma	IL13Ralpha2-specific Hinge-optimized 4-1BB-co-stimulatory CAR/Truncated CD19-expressing Autologous TN/MEM Cells| Ipilimumab Nivolumab	Phase 1	60	26-Sep-19
NCT04099797	C7R-GD2.CAR T Cells for Patients with GD2-expressing Brain Tumors (GAIL-B)	Recruiting	Diffuse Intrinsic Pontine Glioma; High Grade Glioma; Embryonal Tumor; Ependymal Tumor	Genetic: (C7R)-GD2.CART cells Cyclophosphamide Fludarabine	Phase 1	34	3-Feb-20
NCT04214392	Chimeric Antigen Receptor (CAR) T Cells with a Chlorotoxin Tumor-Targeting Domain for the Treatment of MMP2+ Recurrent or Progressive Glioblastoma	Recruiting	Recurrent Glioblastoma; Recurrent Malignant Glioma; Recurrent WHO Grade II Glioma; Recurrent WHO Grade III Glioma	Chlorotoxin (EQ)-CD28-CD3zeta-CD19t-expressing CAR T-lymphocytes (via ICT delivery)<br><br>Chlorotoxin (EQ)-CD28-CD3zeta-CD19t-expressing CAR T-lymphocytes (via ICT/ICV dual delivery)	Phase 1	36	26-Feb-20
NCT05063682	The Efficacy and Safety of Brain-targeting Immune Cells (EGFRvIII-CAR T Cells) in Treating Patients with Leptomeningeal Disease from Glioblastoma. Administering Patients EGFRvIII -CAR T Cells May Help to Recognize and Destroy Brain Tumor Cells in Patients	Active, not recruiting	Glioblastoma; Glioblastoma Multiforme; Glioma, Malignant	BEGFRvIII-specific hinge-optimized CD3 ζ-stimulatory/41BB-co-stimulatory Chimeric Antigen Receptor autologous T-lymphocytes	Phase 1	10	15-May-20
NCT04550663	NKG2D CAR-T(KD-025) in the Treatment of Relapsed or Refractory NKG2DL+ Tumors	Not yet recruiting	Solid Tumor; Hepatocellular Carcinoma; Colorectal Cancer; Glioma	KD-025 CAR-T cells	Phase 1	10	25-Sep-20
NCT05131763	NKG2D-based CAR T-cells Immunotherapy for Patient With r/r NKG2DL+ Solid Tumors	Recruiting	Hepatocellular Carcinoma; Glioblastoma; Medulloblastoma; Colon Cancer	NKG2D-based CAR T-cells	Phase 1	3	1-Mar-21
NCT04661384	Brain Tumor-Specific Immune Cells (IL13Ralpha2-CAR T Cells) for the Treatment of Leptomeningeal Glioblastoma, Ependymoma, or Medulloblastoma	Recruiting	Ependymoma; Glioblastoma; Medulloblastoma; Recurrent Metastatic Malignant Neoplasm in the Leptomeninges	IL13Ralpha2-specific Hinge-optimized 41BB-co-stimulatory CAR Truncated CD19-expressing Autologous T-Lymphocytes	Phase 1	30	5-Mar-21
NCT05241392	Safety and Efficacy Study of Anti-B7-H3 CAR-T Cell Therapy for Recurrent Glioblastoma	Recruiting	Glioblastoma	B7-H3-targeting CAR-T cells	Phase 1	30	27-Jan-22
NCT05474378	B7-H3 Chimeric Antigen Receptor T Cells (B7-H3CART) in Recurrent Glioblastoma Multiforme	Recruiting	Brain and Nervous System	B7-H3CART	Phase 1	39	12-Jul-22

NCT05540873	A Clinical Study of IL13Rα2 Targeted CAR-T in Patients With Malignant Glioma (MAGIC-I)	Recruiting	Recurrent Malignant Glioma	YYB-103	Phase 1	18	18-Jul-22
NCT05366179	Autologous CAR-T Cells Targeting B7-H3 in Recurrent or Refractory GBM CAR.B7-H3Tc	Recruiting	Glioblastoma Multiforme	CAR.B7-H3T cells infusion	Phase 1	36	2-Sep-22
NCT05353530	Phase I Study of IL-8 Receptor-modified CD70 CAR T Cell Therapy in CD70+ and MGMT-unmethylated Adult Glioblastoma (IMPACT)	Not yet recruiting	Glioblastoma	Ex-Vivo expanded autologous IL-8 receptor (CXCR2) modified CD70 CAR (8R-70CAR) T cells	Phase 1	18	Oct-22
NCT05577091	Tris-CAR-T Cell Therapy for Recurrent Glioblastoma	Not yet recruiting	Recurrent Glioblastoma	Inverse correlated dual-target, truncated IL7Ra modified CAR - expressing autologous T-lymphocytes	Phase 1	10	1-Nov-22
NCT05168423	CART-EGFR-IL13Ra2 in EGFR Amplified Recurrent GBM	Not yet recruiting	Glioblastoma	5x10(7) CART-EGFR-IL13Ra2<br>1x107 CART-EGFR-IL13Ra2<br>1x10(8) CART-EGFR-IL13Ra2<br>5x108 CART-EGFR-IL13Ra2<br>Cyclophosphamide<br>Fludarabine	Phase 1	18	19-Mar-23
NCT03638206	Autologous CAR-T/TCR-T Cell Immunotherapy for Malignancies	Recruiting	B-cell Acute Lymphoblastic Leukemia;<br>Lymphoma;<br>Myeloid Leukemia;<br>Multiple Myeloma;<br>Hepatoma;<br>Gastric Cancer;<br>Pancreatic Cance;<br>Mesothelioma;<br>Colorectal Cancer;<br>Esophagus Cancer;<br>Lung Cancer<br>Glioma;<br>Melanoma;<br>Synovial Sarcoma;<br>Ovarian Cancer;<br>Renal Carcinoma	CAR-T cell immunotherapy	Phase 1|Phase 2	73	1-Mar-18
NCT03941626	Autologous CAR-T/TCR-T Cell Immunotherapy for Solid Malignancies	Recruiting	Esophagus Cancer;<br>Hepatoma;<br>Glioma;<br>Gastric Cancer	CAR-T/TCR-T cells immunotherapy	Phase 1|Phase 2	50	1-Sep-19
NCT04077866	B7-H3 CAR-T for Recurrent or Refractory Glioblastoma	Recruiting	Recurrent Glioblastoma;<br>Refractory Glioblastoma	Temozolomide<br>B7-H3 CAR-T	Phase 1|Phase 2	40	1-May-22

Results obtained from clinicaltrials.gov by searching entries with Glioma OR GBM OR Glioblastoma AND CAR-T Therapy and selecting relevant studies (Excluding missing info, unknown status, and pediatric tumors)
